# High-dimensional biomarker identification for interpretable disease prediction via machine learning models

**DOI:** 10.1093/bioinformatics/btaf266

**Published:** 2025-04-26

**Authors:** Yifan Dai, Di Wu, Ian Carroll, Fei Zou, Baiming Zou

**Affiliations:** Department of Biostatistics, University of North Carolina at Chapel Hill, Chapel Hill, NC 27599, United States; Department of Biostatistics, University of North Carolina at Chapel Hill, Chapel Hill, NC 27599, United States; Adams School of Dentistry, University of North Carolina at Chapel Hill, Chapel Hill, NC 27599, United States; Department of Nutrition, University of North Carolina at Chapel Hill, Chapel Hill, NC 27599, United States; Department of Biostatistics, University of North Carolina at Chapel Hill, Chapel Hill, NC 27599, United States; Department of Genetics, University of North Carolina at Chapel Hill, Chapel Hill, NC 27599, United States; Department of Biostatistics, University of North Carolina at Chapel Hill, Chapel Hill, NC 27599, United States; School of Nursing, University of North Carolina at Chapel Hill, Chapel Hill, NC 27599, United States

## Abstract

**Motivation:**

Omics features, often measured by high-throughput technologies, combined with clinical features, significantly impact the understanding of many complex human diseases. Integrating key omics biomarkers with clinical risk factors is essential for elucidating disease mechanisms, advancing early diagnosis, and enhancing precision medicine. However, the high dimensionality and intricate associations between disease outcomes and omics profiles present substantial analytical challenges.

**Results:**

We propose a high-dimensional feature importance test (HiFIT) framework to address these challenges. Specifically, we develop an ensemble data-driven biomarker identification tool, Hybrid Feature Screening (HFS), to construct a candidate feature set for downstream machine learning models. The pre-screened candidate features from HFS are further refined using a computationally efficient permutation-based feature importance test employing machine learning methods to flexibly model the potential complex associations between disease outcomes and molecular biomarkers. Through extensive numerical simulation studies and practical applications to microbiome-associated weight changes following bariatric surgery, as well as the examination of gene-expression-associated kidney pan-cancer survival data, we demonstrate HiFIT’s superior performance in both outcome prediction and feature importance identification.

**Availability and implementation:**

An R package implementing the HiFIT algorithm is available on GitHub (https://github.com/BZou-lab/HiFIT).

## Introduction

High-dimensional omics data, such as genomics, proteomics, and other types of biomedical data generated from high-throughput technologies, have revolutionized clinical research and personalized medicine by providing detailed molecular profiles of individuals (Meyer *et al.* 2013, Ibrahim *et al.* 2016). Omics data offer complementary patient information in addition to low-dimensional baseline demographic and clinical features. This information helps healthcare professionals gaining a deep understanding of the genetic and molecular mechanisms underlying complex human diseases, enabling improved early disease diagnoses and effective personalized treatment strategies tailored to individual patients or subpopulations (Issa *et al.* 2014). However, accurately predicting disease outcomes remains highly challenging due to complex disease mechanisms, including nonlinear impacts of molecular biomarkers and clinical features on disease outcomes, as well as interactive effects among these risk factors. Consequently, conventional parametric methods, such as multiple linear or logistic regression, prove ineffective in constructing powerful predictive models and identifying clinically meaningful molecular biomarkers.

Machine learning algorithms, particularly deep neural networks (DNNs, LeCun *et al.* 2015), support vector machines (SVMs, Cortes and Vapnik 1995), random forests (RFs, Breiman 2001), and gradient boosting machines such as XGBoost (Chen and Guestrin 2016) have demonstrated potential in robustly handling the intricate associations between molecular biomarkers, biological, and clinical features, and disease outcomes (Zhu *et al.* 2020, Poirion *et al.* 2021). Despite powerful and flexible, these models are complicated by the high-dimensionality of multi-omics data, commonly referred to as the “curse of dimensionality” (Mirza *et al.* 2019, Reel *et al.* 2021). Predictive models with high-dimensional input features are susceptible to overfitting, which often results in high training accuracy but poor testing performance. As a result, directly incorporating omics features alongside conventional clinical variables may not enhance disease outcome predictions, even with robustness techniques like pruning, dropout (Hinton *et al.* 2012), and bootstrap bagging and scoring (Mi *et al.* 2019). In addition, interpreting machine learning models trained on high-dimensional multi-omics data presents another significant challenge. Though there are existing methods to identify important biomarkers using machine learning methods, such as the Permutation Feature Importance Test (PermFIT, Mi *et al.* 2021), Shapley Additive Explanations (SHAP, Lundberg and Lee 2017), and Knock-off Randomized Testing (Candès *et al.* 2018), all struggle with the curse of dimensionality. They are limited in their ability to differentiate true important features from noise features when the data dimension is high.

To address the dimensionality issue for omics data integration, researchers extensively utilized shrinkage linear or generalized linear models. These include methods such as the least absolute shrinkage and selection operator (Lasso, Tibshirani 1996) and elastic nets (Zou and Hastie 2005). While powerful, these shrinkage algorithms suffer from performance degradation as the number of nuisance input features increases. Consequently, feature pre-screening becomes necessary. One such approach is Sure Independence Screening (SIS) (Fan and Lv 2008), which selects features with strong marginal effects from high-dimensional data before applying more refined analyses like Lasso (Fan and Lv 2008, Fan and Song 2010). Evaluating the marginal association between each input feature and a disease outcome can be done using metrics such as mutual information (MI), Spearman correlation (SPC), maximal information coefficient (MIC, Reshef *et al.* 2011), or Kendall rank correlation coefficient (Kendall’s tau). For capturing nonlinear associations, researchers may consider methods like the Hilbert-Schmidt independence criterion (HSIC Gretton *et al.* 2007) and kernelized partial correlation (KPC, Huang *et al.* 2022). For multi-omics applications, many existing differential expression (DE) or differential abundance (DA) analysis tools (Love *et al.* 2014, Finak *et al.* 2015) can be naturally incorporated into the SIS framework.

Despite significant advances, several challenges persist. First, the marginal screening approaches mentioned earlier are constrained by individual criteria for measuring marginal dependency, and their performance is often data-dependent. Parametric methods, constrained by assumptions about data-generating models—such as Gaussian or log-Gaussian—may not be suitable for all multi-omics data. For instance, popular parametric DE methods for genomics data may not generalize well to metatranscriptomics or microbiome data (Cho *et al.* 2023). While non-parametric methods relax these distributional assumptions, they can be less effective at capturing simple relationships, such as linear correlations, and no single screening method consistently outperforms others in all cases (Zhang 2019). This, along with the challenges of validating marginal dependency methods in real-world applications (Rapaport *et al.* 2013, Wu *et al.* 2015, Cho *et al.* 2023), makes selecting an appropriate dependency criterion for multi-omics datasets difficult for researchers. Second, integrating omics data with low-dimensional clinical features—such as treatment, medication, or disease history—requires an analysis of interactive effects. However, classical methods like SIS, DE, and DA are designed to measure marginal associations rather than conditional effects, limiting their applicability in these contexts. Third, determining cutoffs for significant omics features can be ambiguous, especially for non-parametric methods. Although non-parametric approaches excel at uncovering novel, nonlinear associations between multi-omics and disease phenotypes, the absence of robust statistical testing and feature validation can lead to significant false discoveries.

To address these challenges, we propose an efficient Hybrid Feature Selection (HFS) framework that combines multiple dependency metrics. HFS identifies important biomarkers by assembling metrics, minimizing the risk of missing important features by relying only on one specific dependency measure. To capture interactions between omics and clinical features, HFS can be easily extended to assess partial associations between disease outcomes and omics traits, conditioned on clinical variables. Additionally, we introduce a novel data-driven method that uses the isolation forest algorithm (Liu *et al.* 2008) to determine the optimal cutoff for dependency statistics, enabling principled identification of important features to boost feature pre-screening performance. While HFS significantly filters out many nuisance biomarkers, it inevitably selects some noise features due to its marginal screening nature. Therefore, a further refinement process is necessary to fine-tune the HFS list and determine the most relevant features for outcome prediction. Furthermore, it is critically important to evaluate the impact of each individual pre-selected biomarker on disease outcomes by adjusting for the confounding effects of other biomarkers under complex association settings. This can not only deeper our understanding of disease mechanisms but also informs better clinical decisions. To achieve this objective, we leverage PermFIT (Mi *et al.* 2021), a computationally efficient framework to evaluate each individual pre-screened feature’s impact on disease outcome with a rigorous statistical inference under potential complex associations for machine learning models. Combining PermFIT with HFS, we alleviate the curse of dimensionality, allowing more effective detection of complicated impacts such as nonlinear interactions among omics features on disease outcomes. We consolidate the entire process into a comprehensive high-dimensional feature importance test (HiFIT) framework. This framework encompasses feature pre-screening, refinement, and final predictive modeling, achieving robust, scalable, and interpretable disease outcome predictions in high-dimensional settings as delineated in the following section.

## Materials and methods

The proposed HiFIT framework comprises two main components: feature pre-screening by HFS and machine learning-based feature importance testing using the PermFIT algorithm (Mi *et al.* 2021). HFS pre-screens high-dimensional multi-omic features by evaluating their complex marginal association with the outcome, addressing the curse of dimensionality. Following pre-screening, the selected high-dimensional features, together with low-dimensional clinical variables, are incorporated into machine learning models—such as DNN, RF, XGBoost, and SVM—to develop initial predictive models. PermFIT then assesses the impact of each HFS-selected feature on the outcome under intricate associations. This helps to further filter out unimportant features, control the false discoveries, and boost prediction performance.

### HFS: hybrid feature screening

Let $y={\left(y_{1},\ldots ,y_{n}\right)}^{T}$ be the clinical outcome of interest across *n* samples, and $X=\{x_{ij}\}$ be an n×p matrix, where $x_{ij}$ is the $j^{th}$ feature of sample *i*, and *p* is the number of input features. Further, define $x_{i\cdot }={\left(x_{i1},\ldots ,x_{ip}\right)}^{T}$ and $x_{\cdot j}={\left(x_{1j},\ldots ,x_{nj}\right)}^{T}$, and assume that among the *p* input features, there exists an important feature subset *S* with |S|<p such that $E[y_{i}|x_{i\cdot }]=E[y_{i}|\{x_{ij},j\in S\}]$. To ensure the selection power of important features, we follow the sparsity assumption of SIS such that the true number of important features |S|≪p. We also follow SIS to refer the important features as important features while the other features are referred to as noise features. In contrast to SIS, HFS screens important features whose effects on the outcome are linear or more complex than linear by combining (i) parametric utility metrics, such as statistics in generalized linear models commonly used in DE analysis (Finak *et al.* 2015), and (ii) non-parametric utility metrics like kernel-based correlations. In this article, we set the utility metrics to (i) the adjusted R-squared for polynomial regression and (ii) the kernel partial correlation (KPC, Huang *et al.* 2022) coefficient for HFS. To determine a cutoff for screened features, HFS leverages the isolation forest algorithm (Liu *et al.* 2008), which assigns an anomaly score to each feature, with higher scores indicating stronger associations with the outcome.

To compute the parametric utility function, we fit the following linear or generalized linear model independently for each feature (say $j^{th}$ feature),


(1) $$E[y_{j}|x_{ij}]=g^{-1}(\beta_{0}+\sum_{m=1}^{M}\beta_{m}x_{ij}^{m})\;$$

with an appropriate link function *g* corresponding to the outcome type, for example, g(x)=x for continuous outcome, and g(x)=logit(x) for binary outcome, and a pre-specified order *M* polynomial. The adjusted R-square or McFadden’s pseudo R-square (McFadden 1974), denoted as $\rho_{j1}$, is computed as follows:


$$\rho_{j1}=\{\begin{array}{c} 1-\frac{n-1}{n-M-1}\left[\frac{\sum_{i=1}^{n}{\left(y_{i}-{\hat{y}}_{i}\right)}^{2}}{\sum_{i=1}^{n}\left(y_{i}-{\bar{y}}^{2}\right)}\right],\;\text{for}\;\text{continuous}\;\text{outcome},\\ 1-\frac{LL_{j}}{LL_{0}},\;\text{for}\;\text{binary}\;\text{outcome}, \end{array}$$

where $\bar{y}$ is the sample mean of the outcome, and ${\hat{y}}_{i}$ is the predicted outcome of sample *i*; $LL_{j}$ is the maximal log-likelihood of model (1), and $LL_{0}$ is the maximal log-likelihood of the null model with an intercept only. In our analysis, in line with many parametric DE/DA methods, we search for the linear and quadratic associations by setting *M* to 2.

For more general marginal non-linear association detection, a natural extension of model (1) involves increasing *M* to capture associations in higher orders, potentially extending to infinite moments. Alternatively, we adopt the KPC coefficient of each feature within the Reproducing Kernel Hilbert Space (RKHS) framework (Huang *et al.* 2022) to approximate the infinite extension of model (1), where the n×n sample kernel matrices are defined as:


$${\left(K_{j}\right)}_{kl}=k(x_{kj},x_{lj}),\;{\left(K_{y}\right)}_{kl}=k(y_{k},y_{l})\;$$

with κ being a kernel function. The centered kernel matrices are denoted as $\tilde{K_{j}}=HK_{j}H$ and $\tilde{K_{y}}=HK_{y}H$ where $H=I-\frac{1}{n}11^{T}$. Here, *I* denotes an *n* by *n* identity matrix, and 1 denotes a 1-vector of size *n*. The empirical KPC between *y* and the $j^{th}$ feature is:


(2) $${\hat{\rho }}_{j2}=\frac{\text{tr}(O_{j}^{T}\tilde{K_{y}}O_{j})}{\text{tr}(\tilde{K_{y}})},$$

where $O_{j}=\tilde{K_{j}}{\left(\tilde{K_{j}}+n\delta I\right)}^{-1}$ with δ being a positive constant. Notably, if a linear kernel $\kappa (a,b)=a^{T}b$ is used, the KPC coefficient in Equation (2) approximates the adjusted R-square of model (1) when M=1. Higher-order variations of model (1) can be approximated using KPC with the corresponding polynomial kernel. In our analysis, we employ an infinite-dimensional kernel, or the radial basis function kernel $\kappa (a,b)=\text{exp}\;\{||a-b|{|}_{2}^{2}/2\}$ to flexibly detect complex omics-phenotype associations.

Both utility functions have their limitations. First, model (1) with lower orders may fail to capture complex higher-order associations, while KPC is less effective at detecting linear associations. Second, model (1) allows for the use of a likelihood ratio test to derive a p-value, providing a clear criterion for significance. In contrast, the value of the utility function (2) is more difficult to interpret, making it challenging to determine an appropriate cutoff for significance. To address the first challenge, HFS combines the two utility functions of the $j^{th}$ feature into a correlation vector, $\rho_{j}={\left(\rho_{j1},\rho_{j2}\right)}^{T}$ to generate a biomarker list highly correlated with the disease outcomes, referred to as the HFS list. To address the second challenge, and recalling the sparsity assumption in SIS, we model the distribution of $\rho_{j}$s through a mixture model. In this model, we assume that the vector corresponding to the noise features forms one cluster with a distribution of $g_{0}$, while the vector associated with the remaining ones forms another cluster with a distribution of $g_{1}$. More specifically, we have


$$\rho_{j}∼\pi g_{0}+(1-\pi )g_{1},\;j=1,\ldots ,p$$

where π is the proportion of the noise features. The cumulative distribution function of $g_{0}$, $G_{0}(x)$, is assumed to be no smaller than $G_{1}(x)$, the cumulative distribution function of $g_{1}$. The features classified to the second cluster are considered anomaly features which are expected to overlap largely with set *S* as long as the marginal utilities of the important features are not too small. We utilize the isolation forest (Liu *et al.* 2008), a bootstrapping ensemble of isolation trees to estimate the anomaly probability of each feature as follows: for *B* bootstrap replicates, the anomaly score of feature *j* is given by


$$s(\rho_{j},\psi )=2^{-\frac{1}{B}\sum_{b=1}^{B}h^{\left(b\right)}(\rho_{j})/c(\psi )}\in [0,1]$$

where ψ is the bootstrapped sample size regularized by c(ψ), and $h^{\left(b\right)}(\cdot )$ represents the path length from the root to the leaf node that the sample belongs to, adjusted by the average path length of the $b^{th}$ isolation tree. For a given cutoff τ, the selected feature set is given by


(3) $$\hat{S}:=\left\{j:s(\rho_{j},\psi )\geq \tau \right\}.$$

To integrate multi-omics features with low-dimensional clinical data, it is important to model their interactive effects. For example, specific omics features may interact with treatments, influencing disease outcomes and enabling personalized medicine. To capture these interactions, we can replace the default utility functions in HFS with their conditional counterparts that measure if certain omics traits are correlated with the disease outcomes given the level of clinical features. This adjustment will allow the HFS framework to identify a greater number of features that interact with clinical variables. Further details on this modification are available in the Supplementary Section S1.

### Machine learning based feature importance test

To provide quality checking and formal statistical testing for features in the HFS list, HiFIT employs the PermFIT procedure, a permutation-based feature importance score test originally developed for seemingly black-box machine learning models (Mi *et al.* 2021). Suppose y=f(x)+ϵ, and $\hat{f}(\cdot )$ is the estimated function of f(·) from a machine learning model. While HFS ensures that the biomarkers x are marginally correlated with the disease outcome *y—*such as genes that are differentially express—the f(·) can model the conditional effect of each biomarker given other elements in x. Thus, HiFIT can refine the HFS lists by accounting for more confounding structures within x. In the remaining part, we outline the PermFIT procedure to derive feature-wise p-values. Notably, we use some notation shortcuts and assume that the input feature vector now contains only the features in the HFS list $\hat{S}$. Consequently, *p* now refers to the size of $\hat{S}$, which is much smaller than the original number of high-dimensional input features. In this article, we focus on four popular machine learning algorithms, although the framework is not limited to them: (i) SVM—implemented via R package “e1071” using Radial kernels, (ii) RF—implemented via R package “randomForest”, (iii) XGBoost—implemented via R package “xgboost”, and (iv) ensemble DNN—implemented via R package “deepTL” (Mi *et al.* 2019).

For the $j^{th}$ feature in $\hat{S}$, we define its importance score $\Lambda_{j}$ as $\Lambda_{j}=E_{x_{i,\cdot },x_{i}^{\left(j\right)}}\left[{\left\{f(x_{i.})-\hat{f}(x_{i}^{\left(j\right)})\right\}}^{2}-{\left\{f(x_{i.})-\hat{f}(x_{i\cdot })\right\}}^{2}\right],$ where $x_{i}^{\left(j\right)}={\left(x_{i,1},\ldots ,x_{i,j}^{\mathrm{permuted}},\ldots ,x_{i,p}\right)}^{T}$ with $x_{i,j}^{\mathrm{permuted}}$ being the $i^{th}$ element of the permuted vector of $x_{\cdot j}$. The importance score $\Lambda_{j}$ equals zero when the contribution of the $j^{th}$ feature to *y* is null, and that, the stronger the impact of the $j^{th}$ feature on the outcome, the larger $\Lambda_{j}$ is. PermFIT then empirically estimates $\Lambda_{j}$ by ${\hat{\Lambda }}_{j}=\frac{1}{n}\sum_{i=1}^{n}\left[{\left\{y_{i}-\hat{f}(x_{i}^{\left(j\right)})\right\}}^{2}-{\left\{y_{i}-\hat{f}(x_{i\cdot })\right\}}^{2}\right]$. To avoid potential overfitting of *f* for data with finite samples, PermFIT employs a data splitting strategy in which it divides the data into training and validation sets. It uses the training set for generating $\hat{f}(\cdot )$ and the validation set for evaluating the distribution of $\Lambda_{j}$. That is, let ${\hat{f}}_{T}(\cdot )$ denote the estimate of f(·) from a training set, and $\Omega_{V}={\left\{y_{i},x_{i\cdot }\right\}}_{i=1}^{n_{V}}$ be the validation set, we obtain the feature importance score estimate ${\hat{\Lambda }}_{j}$, and its associated variance as ${\hat{\Lambda }}_{j}=\frac{1}{n_{V}}\sum_{i=1}^{n_{V}}{\hat{\Lambda }}_{ij}$ and $\hat{\text{Var}}[{\hat{\Lambda }}_{j}]=\frac{1}{n_{V}}\sum_{i=1}^{n_{V}}{\left[{\hat{\Lambda }}_{ij}-{\hat{\Lambda }}_{j}\right]}^{2}$, respectively, with ${\hat{\Lambda }}_{ij}=\frac{1}{n_{V}}\sum_{i=1}^{n_{V}}\left[{\left\{y_{i}-{\hat{f}}_{T}(x_{i}^{\left(j\right)})\right\}}^{2}-{\left\{y_{i}-{\hat{f}}_{T}(x_{i\cdot })\right\}}^{2}\right]$. The statistics for feature importance test of $X_{j}$ can be constructed as $\lambda_{j}=\frac{{\hat{\Lambda }}_{j}}{\sqrt{\hat{\text{Var}}[{\hat{\Lambda }}_{j}]}}∼N(0,1)$ under the null. With the proposed test, $\hat{S}$ is further refined to a final feature list, denoted as ${\hat{S}}_{\mathrm{final}}$ and used by HiFIT to build the final predictive model. The importance score test for the binary outcome can be constructed in a similar manner, and the details can be found in Mi *et al.* (2021).

### Adaptive selection of the HFS cutoff parameter

In this section, we propose a data-driven approach that heuristically and computationally efficiently searches for an optimal τ from a set of candidates (ordered from the smallest to the largest) $\left(\tau_{0},\tau_{1},\ldots ,\tau_{R}\right)$, with $\left\{{\hat{S}}_{1},\ldots ,{\hat{S}}_{R}\right\}$ being the corresponding selected feature sets. These sets consist of features with their anomaly scores falling in the intervals of the candidate cutoffs. That is,


$${\hat{S}}_{r}:=\{j:s(\rho_{j},\psi )\in [\tau_{r-1},\tau_{r})\},\;r=1,\ldots ,R.\;$$

For the $r^{th}$ feature sets, we estimate its set importance score $\Lambda_{S_{r}}=E_{x_{S},x^{\left(S_{r}\right)}}{\left[f(x_{S})-f(x^{\left(S_{r}\right)})\right]}^{2}$, analogous to the way that PermFIT defines feature importance score, where $x_{S}$ denotes the features with the HFS anomaly score larger than $\tau_{0}$, and $x^{\left(S_{r}\right)}$ is a rearranged $x_{S}$ with features in $S_{r}$ replaced by random permutations. For computational efficiency, instead of testing the importance of each feature, we estimate the set importance scores here. The smallest τ among all candidates, for which the feature sets have a *P*-value smaller than 0.1 ($p_{\left(S_{r}\right)}<0.1$), will be selected by HiFIT as the final cutoff. In this article, we set the candidate list as (0.5,0.55,0.6,0.65,0.7) for both simulation studies and real data analysis.

## Results

To evaluate the performance of HiFIT in eliminating nuisance features and predicting outcomes, we conducted comprehensive simulation studies under various data complexity scenarios with varying numbers of features and data generation schemes. For comparison, we included the gold standard approach, which uses only important features as input, alongside Lasso and other machine learning algorithms. Additionally, HiFIT was applied to two real-world datasets: the weight loss data after bariatric surgery (BS) (Heinberg *et al.* 2020, Fouladi *et al.* 2021), and RNA sequence data from three kidney cancer studies in The Cancer Genome Atlas (TCGA, Cancer Genome Atlas Research Network *et al.* 2013). Given the unknown biological ground truth of the associations between clinical and omics features and disease phenotypes, we evaluated the performance of each comparison method by focusing on the prediction accuracy of the final HiFIT model in real data applications.

### Simulation studies

We examine the performance of the proposed methods under the following simulation scenarios: (a) **Linear case**: where the effects of the important features are linear and additive; (b) **Non-linear case**: where the effects of the important features are non-linear and non-additive. Specifically, we simulate *y* as follows:


$$\begin{array}{c} y∼\sum_{j=1}^{10}\beta_{j}x_{j}+\epsilon \;\;\text{and}\;\\ y∼\sum_{j=1}^{4}2\;\text{sin}(2x_{j})-\sum_{j=5}^{8}2\;\text{log}(2x_{j}^{2}+1)+x_{9}\;\text{exp}(x_{10})+\epsilon \end{array}$$

for cases (a) and (b) respectively, where $x={\left(x_{1},\ldots ,x_{p}\right)}^{T}$ is a p-dimensional random variable drawn from a multivariate normal distribution, multinomial distribution, or Poisson log-normal distribution with block correlation, the error term ϵ follows a standard normal distribution, that is, N(0,1), and the vector $\beta =(\beta_{1},\ldots ,\beta_{10})$ is drawn from a uniform distribution U(1,1.5) at the beginning of the simulation and remains unchanged throughout subsequent Monte Carlo simulations. For both cases, we set the total number of important features to 10, with the remaining p−10 features being nuisance variables. The total number of samples is set to 500. To evaluate the impact of input feature dimensions, we vary *p* across the values {500, 1000, 10 000}. We split the simulated data into training and testing sets in a 9:1 ratio. For each scenario, the total number of simulation is set to 100. Since the performance of HFS and HiFIT is similar across different distributions of x, we primarily present the results with x drawn from multivariate normal distributions and leave remaining results in the Supplementary Figs S1–S6.

**Figure 1.**
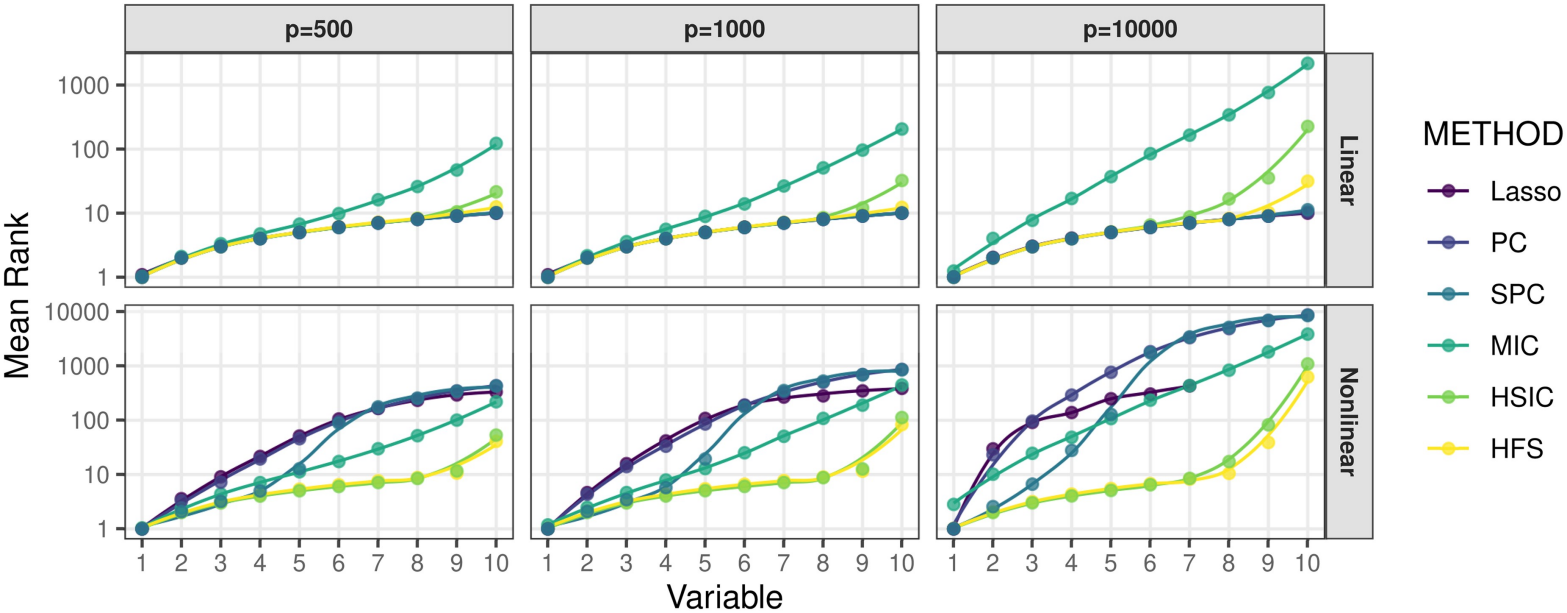
Average rank of important features selected by pre-screening methods. The *x*-axis denotes the number of selected important features, and the corresponding value of the *y*-axis represents the average rank of this feature over 100 repetitions. The curves are generated by locally estimated scatterplot smoothing.

We begin by comparing the performance of HFS with other feature pre-screening methods, including Lasso, Pearson Correlation (PC), Spearman Correlation (SPC), MIC, and HSIC. Figure 1 illustrates the ranks of correlation scores for important features relative to all features. Since Lasso does not directly produce a correlation score, we rank the penalty parameter associated with the important features. Specifically, we consider the largest penalty parameter at which the coefficient of a feature becomes non-zero. Under linear settings, when p=500 or 1000, HFS performs comparably to parametric models like Lasso and PC in ranking important features. As the dimensionality *p* increases to 10 000, HFS still outperforms non-parametric methods such as HSIC and MIC. Notably, HSIC is significantly influenced by noisy variables and ranks half of the important features with smaller linear effects much lower than HFS. The advantage of HFS becomes even more prominent in non-linear scenarios. Parametric methods like Lasso and PC become ineffective and fail to identify important features, as shown in the second panel of Fig. 1. On the other hand, while SPC and MIC relax the parametric assumptions of Lasso and PC, they only rank half of the important features at the top. Specifically, SPC struggles to detect monotonic associations, while MIC tends to capture spurious correlations between nuisance features and outcomes. Furthermore, their performance deteriorates as the dimensionality increases to 10,000. In contrast, HFS not only identifies the largest number of important features but also remains robust in high-dimensional data scenarios. In summary, HFS effectively combines the advantages of both parametric and non-parametric methods for feature screening by leveraging different marginal association dependencies. Other methods are either constrained by their parametric assumptions or limited in their ability to handle high-dimensional data.

**Figure 2.**
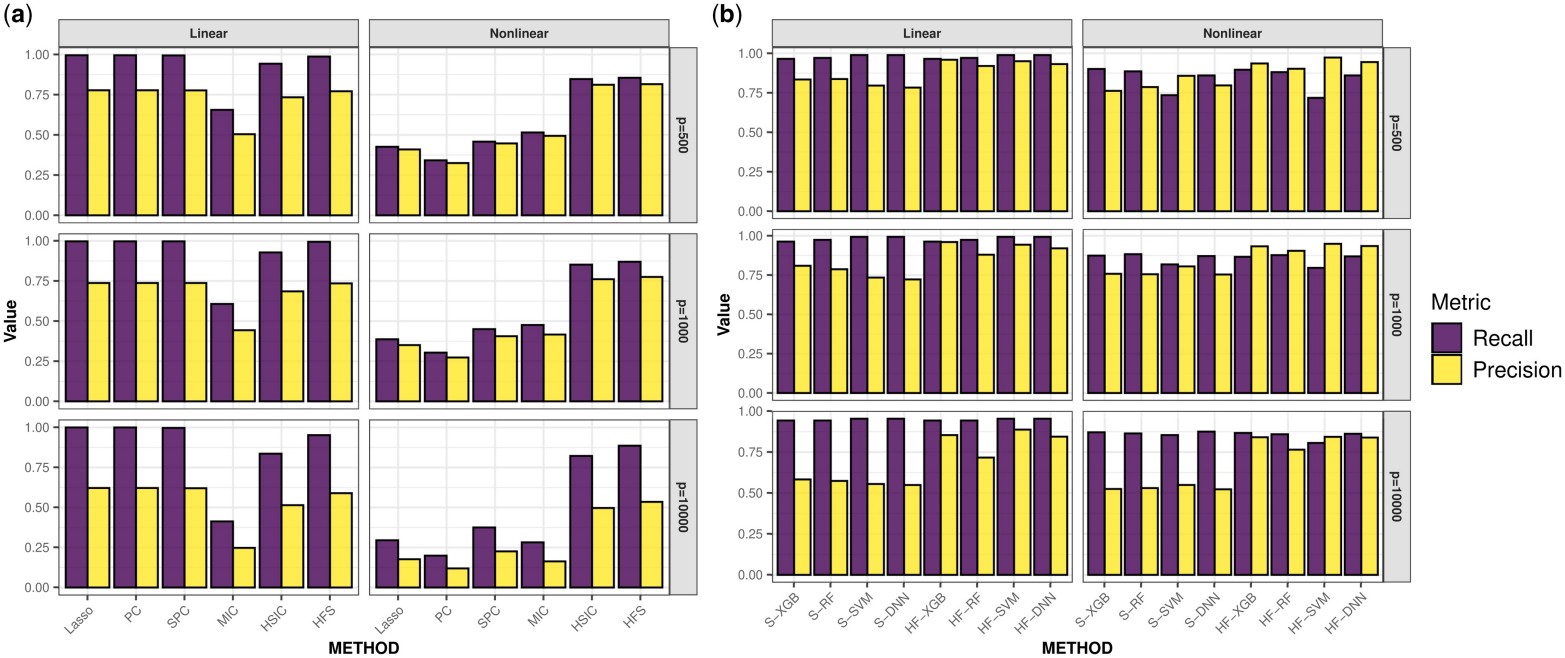
High-dimensional feature pre-screening and selection results. (a) Performance of feature pre-screening methods. (b) Feature selection results of HiFIT models. Recall and precision are averaged over 100 simulations.

In practice, the cutoff for the HFS score is determined using a data-driven approach (see section Materials and methods). Features with HFS scores higher than the cutoff are retained for downstream analysis. Since many comparison methods do not provide a data-driven cutoff, we select the same number of features as HFS across all comparison methods to ensure a fair comparison. We evaluate the quality of the selected feature list based on recall and precision. Recall is defined as the ratio of selected important features to the total number of important features, while precision is defined as the ratio of selected important features to the total number of selected features. Figure 2a illustrates the quality of feature sets. HSIC and MIC tend to overlook important features with smaller effects under linear settings. Meanwhile, Lasso, PC, and SPC struggle to detect log-quadratic or interaction terms. In comparison, HFS consistently identifies more important features across all simulation scenarios. However, as the dimensionality increases, the precision of all pre-screening methods decreases. To address this issue, HiFIT refines the pre-screening feature set obtained from HFS using machine learning algorithms. Figure 2b compares (i) HFS feature sets with the cutoff parameter determined by XGB, RF, SVM, and DNN (denoted as S-XGB, S-RF, S-SVM, and S-DNN); (ii) HiFIT feature sets obtained by applying PermFIT to the corresponding HFS feature lists, retaining features with p-values smaller than 0.1 (denoted as HF-XGB, HF-RF, HF-SVM, and HF-DNN).

HiFIT feature sets retain most of the important features identified by HFS. Specifically, the recall of HF-XGB, HF-RF, and HF-DNN match those of the corresponding HFS feature sets, and the recall of HF-SVM is comparable to S-SVM. Furthermore, HiFIT improves the precision of HFS. For instance, when p=500 or 1000, the precision of HF-XGB, HF-SVM, and HF-DNN is controlled at 0.9. As *p* increases to 10 000, HF models improve the precision of the corresponding HFS sets from 0.5 to more than 0.8, and further reductions are possible with *p*-value adjustments.

**Figure 3.**
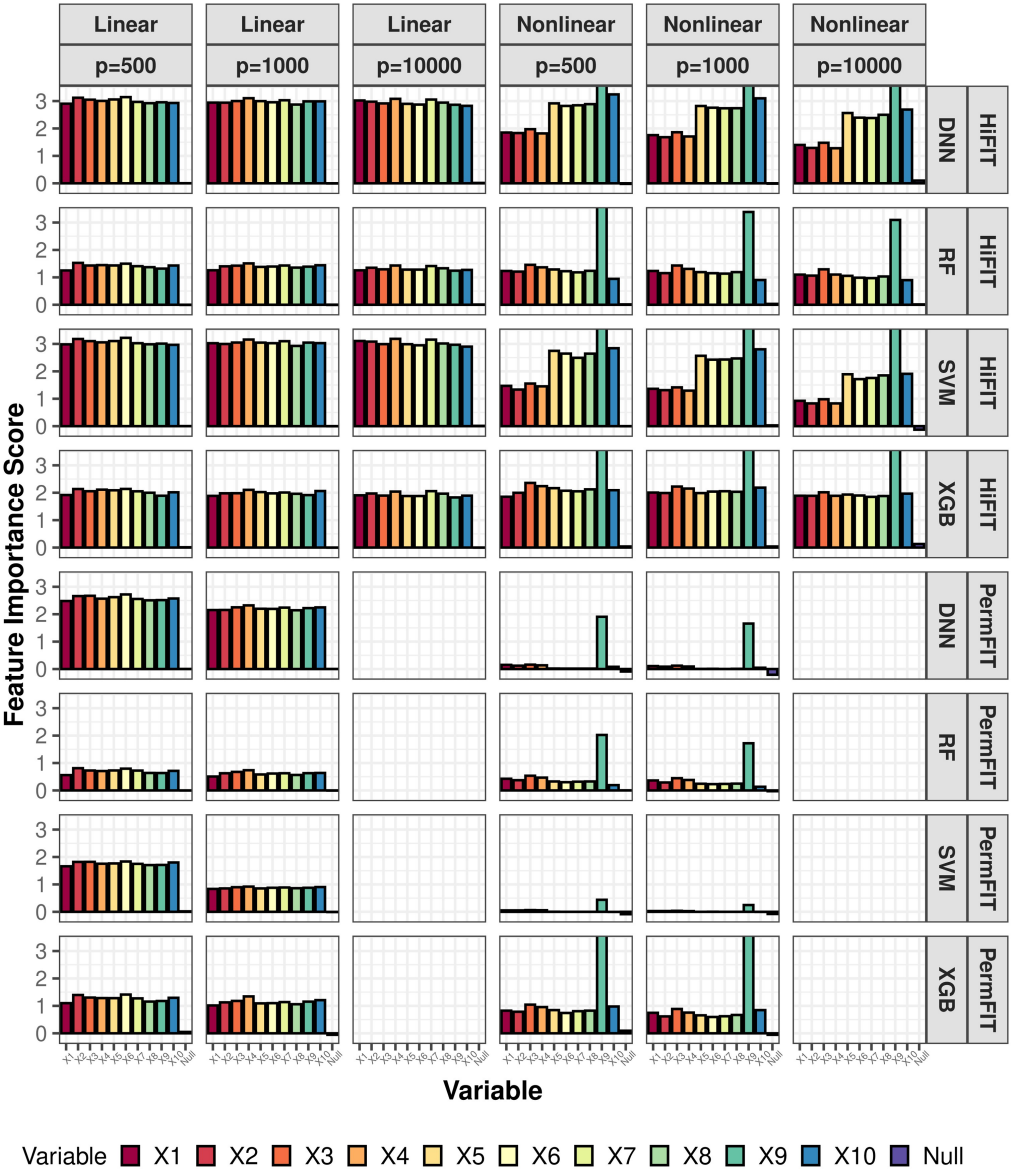
HiFIT feature interpretation results. Average feature importance scores for 10 important features (denoted as $X_{1},\ldots ,X_{10}$) and the feature set of nuisance features (denoted as null) over 100 repetitions. Importance scores of features not selected by HFS are set to zero.

Next, we compare the importance scores and *p*-values from HiFIT with those from PermFIT. Figure 3 provides detailed feature importance scores for the following scenarios: (i) HiFIT models: HF-DNN, HF-SVM, HF-XGB, and HF-RF; and (ii) PermFIT models: PermFIT-DNN, PermFIT-SVM, PermFIT-SVM, and PermFIT-XGB (applied to the same models without pre-screening). We observe that all HiFIT models successfully identify important features, consistently estimating high importance scores for these features across various simulation scenarios. HiFIT assigns low importance scores to nuisance features, demonstrating its ability to control type-I errors reasonably in high-dimensional settings, regardless of association complexity. In contrast, PermFIT models struggle with nonlinear effects. PermFIT-DNN and PermFIT-SVM identify only one nonlinear important feature out of ten on average. While PermFIT-RF and PermFIT-XGB successfully identify all nonlinear features, their importance scores for important features are substantially lower than those from HF-XGB and HF-RF, highlighting HiFIT’s superior efficiency. For instance, the importance score of the interaction term $X_{10}$ from PermFIT-RF is lower than that from HF-RF, indicating that PermFIT-RF is more likely to overlook this feature. As data dimensionality increases, PermFIT’s computation cost becomes overwhelming when p=10 000, whereas HiFIT remains scalable to high-dimensional data.

**Figure 4.**
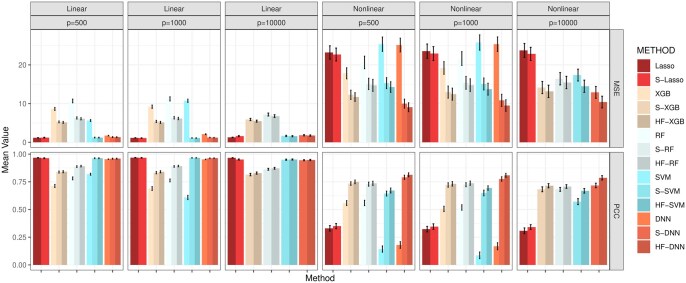
Average MSE and PCC for methods in comparison. Lasso, XGB, RF, SVM, and DNN: specific models with all features; S-Lasso, S-XGB, S-RF, S-SVM, S-DNN: specific models with HFS pre-screening; HF-XGB, HF-RF, HF-SVM, HF-DNN: specific models with HiFIT feature selection. Simulation in each scenario is repeated 100 times.

Finally, we assess the impact of HFS and HiFIT on prediction accuracy for various machine learning models. Figure 4 summarizes the predicted average mean squared error (MSE) and Pearson correlation coefficient (PCC) for the following scenarios: (i) Taking the full features as input: Denote the corresponding models as Lasso, DNN, XGB, RF, and SVM; (ii) Taking the HFS pre-screened features as input: Denote the corresponding models as S-Lasso, S-DNN, S-XGB, S-RF, and S-SVM; (iii) Taking the HiFIT refined features (with P≤.1) as input: Denote the corresponding models as HF-DNN, HF-XGB, HF-RF, and HF-SVM. Under linear settings, and for scenario i), all machine learning models perform worse than Lasso, and their prediction errors increase with the data dimensionality. When P=10 000, RF, DNN, and SVM all fail to converge. After HFS, S-SVM and S-DNN achieve performance comparable to Lasso across dimensions, with similar PCC and MSE. S-RF and S-XGBoost perform slightly worse than Lasso but close to their optimal predictions (using only true important features, see Supplementary Section S2.1). Similarly, under the nonlinear cases, without pre-screening, all four machine learning methods struggle to make reliable predictions (see Fig. 4). Trained with the only 10 important features, SVM and DNN outperform XGBoost and RF, highlighting their better ability in capturing complex feature-outcome relationships when a set of right features are provided. However, in high-dimensional settings with many nuisance features, their performance becomes inferior to RF and XGBoost, and they even become computationally infeasible for P=10 000. In contrast, HFS significantly improves performance of these models, especially for SVM and DNN. S-DNN achieves the lowest prediction error and highest PCC across dimensions, surpassing other machine learning algorithms. Even at P=10 000, S-DNN maintains a high PCC of 0.77. Overall, HFS enhances machine learning models by reducing MSE (by 30%) and increasing PCC (by 20%) for P=500 and 1000. The models remain stable as feature dimension increases, with PCC consistently above 0.7.

In addition to the simulation above, we conducted numerical studies to evaluate the performance of HiFIT for scenarios with non-Gaussian predictors, mixture of linear and nonlinear effects, and binary outcomes (Supplementary Sections S2.2–S2.4). Similar conclusions were observed, and detailed results are presented in the supplementary materials. Beyond the simulation studies, we applied the proposed HiFIT framework to two practical studies, i.e., post BS weight loss and kidney pan-cancer (KIPAN) study, as described below. Similar to the simulation, we randomly split the each real dataset into training and testing sets in a 9:1 ratio and replicate 100 times.

### Weight loss after bariatric surgery study

**Figure 5.**
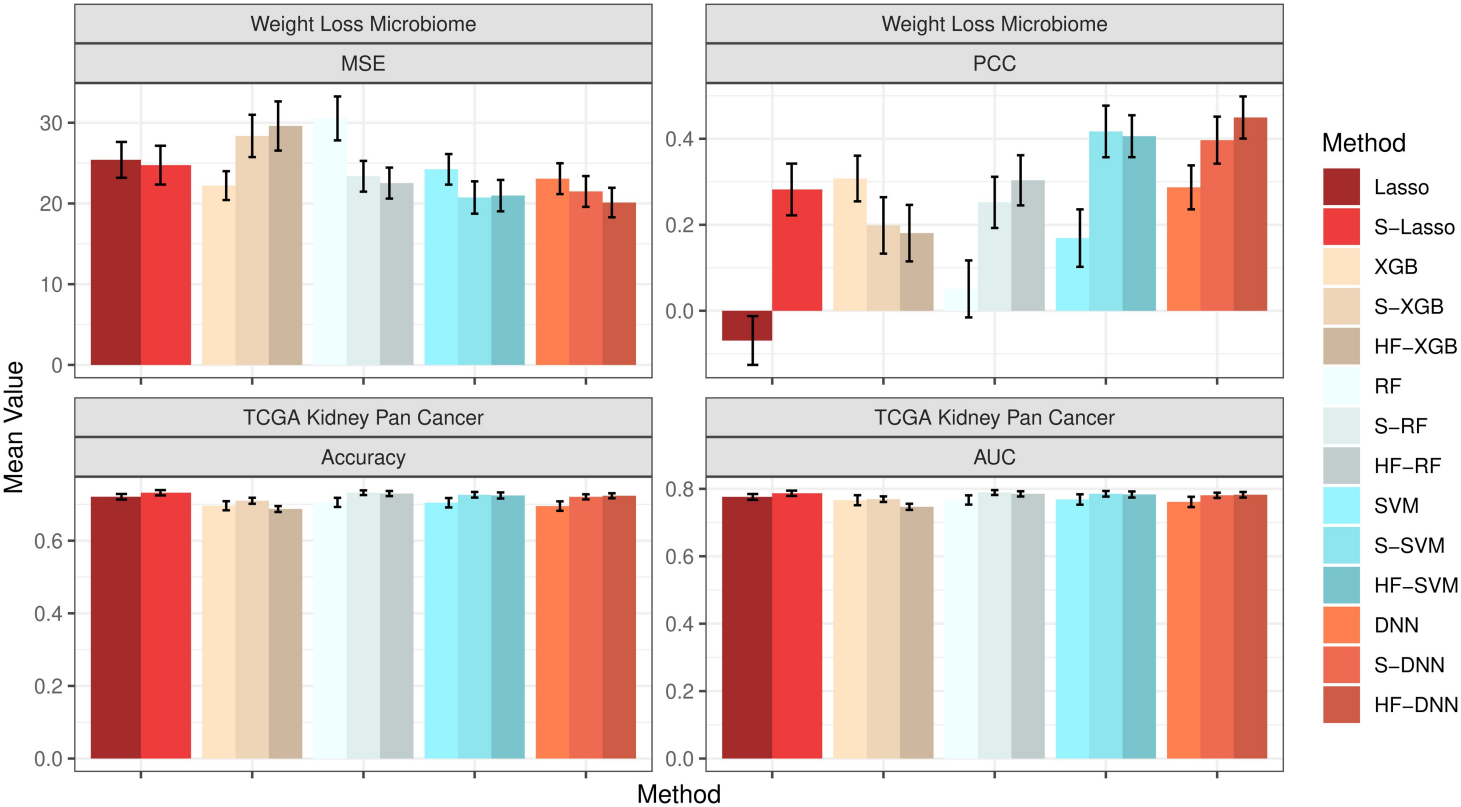
Model performance for the real data analysis. The first column presents the MSE and PCC for the weight loss microbiome study. The second column presents the accuracy and AUC for TCGA kidney pan cancer cohort. All metrics are averaged on separate testing sets consisting of 10% observations with random repeats for 100 times.

In the first real application, we applied the HiFIT to a weight loss study for BS. One of the primary objectives of this study was to use the baseline microbiome profiles along with other demographic and clinical features to predict postoperative weight loss and identify associated important features. The weight loss microbiome cohort consists of 144 participants undergoing BS with 50% of them having Roux-en-Y Gastric Bypass (RYGB) and the other 50% having Sleeve Gastrectomy (SG) (Heinberg *et al.* 2020, Fouladi *et al.* 2021). The body mass index (BMI) and fecal material were collected from individuals at 1, 6, 12, 18, and 24 months post-surgery. The microbial profiles of the BS study were characterized through shotgun Whole Genome Sequencing across multiple time points of 135 subjects (n=135), resulting in total 430 measurements of BMI change with 1533 microbial genera. There are also four demographic features of participants, for example, age, race, height, and sex. In this study, we aim to predict patients’ BMI change from the day of surgery to their last recorded measurement, using their surgery type, time since surgery, demographic features, and the gut microbiome abundance collected from their first visit. Since the effect of the surgery type and demographic features on weight change is of particular interest (Heinberg *et al.* 2020), we retain all these features and only perform pre-screening on the microbiome features with HFS. Figure 5 presents the model performance, demonstrating that HFS substantially enhances the prediction accuracy of Lasso, SVM, RF, and DNN. HiFIT further improves the performance of RF and DNN. Specifically, HF-DNN achieves the highest prediction accuracy among all methods, with the smallest predicted MSE and the largest PCC.

**Figure 6.**
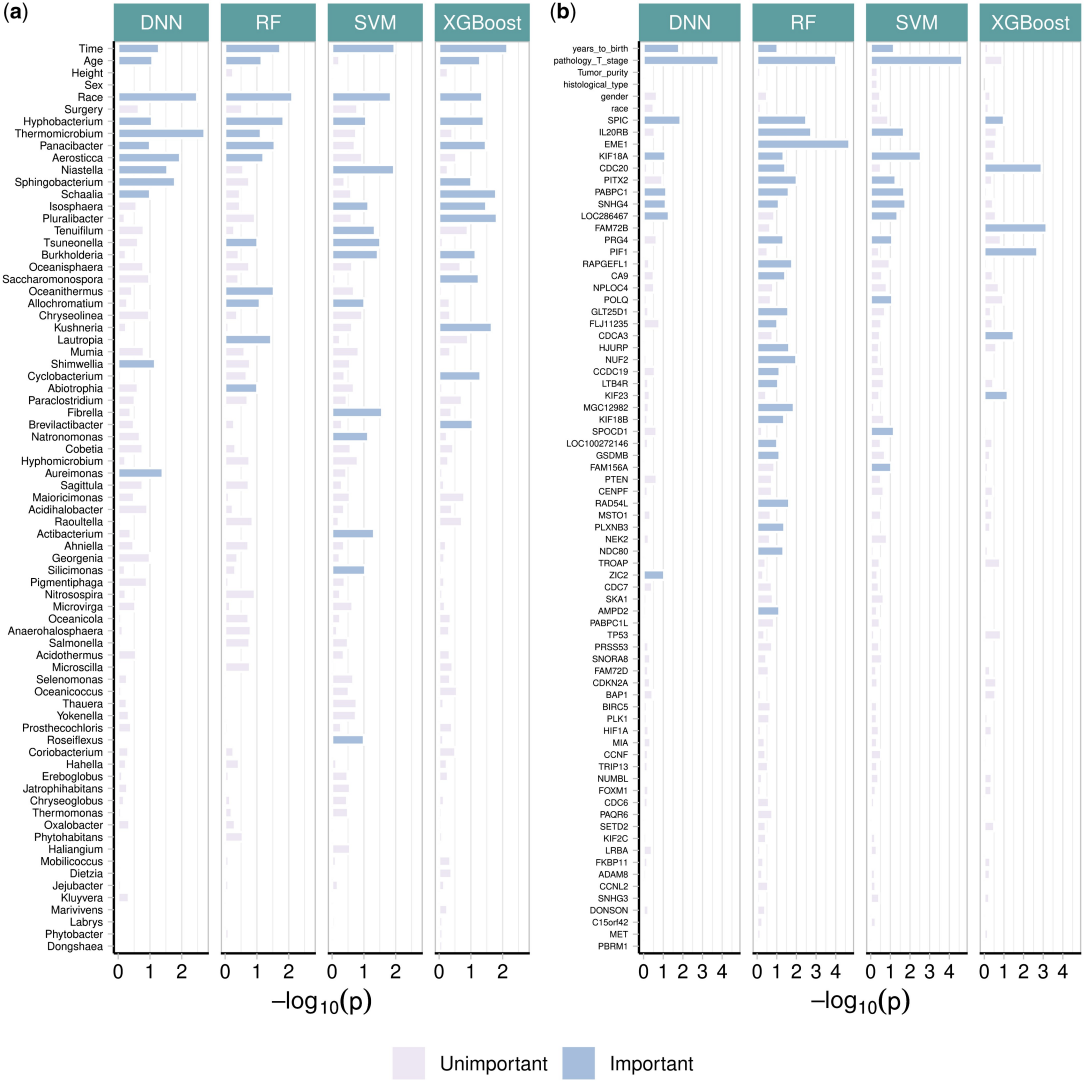
Negative ${\;\text{log}\;}_{10}$  *P*-values for biomarkers from real datasets. (a) Feature importance for the weight loss data. (b) Feature importance for the TCGA data.

**Figure 7.**
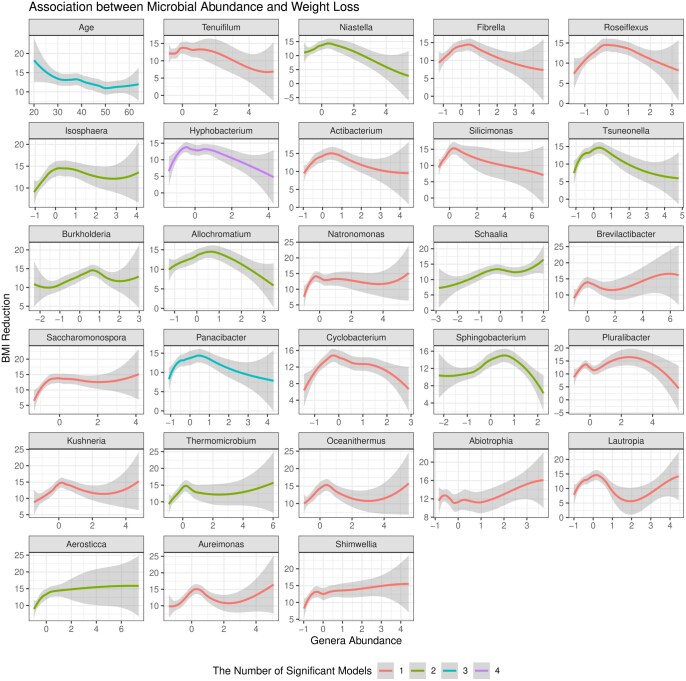
Association between BMI reduction and microbiome abundance. The *x*-axis represents the logarithmic abundance of microbial genera identified by HiFIT in Fig. 6a. The *y*-axis represents the reduction in BMI following bariatric surgery. We also include the association between age and BMI reduction as a reference. The smoothing curves and 95% confidence bands are obtained by locally polynomial regression. The color of the curves represents the number of HiFIT models in Fig. 6a identifying the feature as significant.

Figure 6a presents the feature-wise *P*-values from the four machine learning models in HiFIT. Some demographic features, including time, age, and race, have significant effects on weight loss after BS. The finding on age is consistent with clinical findings (Contreras *et al.* 2013). HiFIT provides further insights into microbial effects on weight loss. As shown in Fig. 6a, all four machine learning models identify important microbiota related to weight loss. This implies that gut microbial abundance offers a distinct source of information on weight loss beyond patients’ demographic features, aligning with previous studies (Zhang *et al.* 2009, Ferrocino *et al.* 2015). Specifically, all HiFIT models identify the beneficial microbe *Hyphobacterium* (Ferrocino *et al.* 2015) as an important predictor for post-BS weight loss, and three HiFIT models highlight *Panacibacter* as an important genus. Figure 7 further illustrates the nonlinear relationship between BMI reduction and microbial abundance. Unlike the approximately linear effect of age on weight loss, most microbial effects exhibit quadratic or even non-polynomial patterns, underscoring the need for nonlinear feature screening and flexible modeling using machine learning methods included in HiFIT. Moreover, the effects of the two aforementioned genera, *Hyphobacterium* and *Panacibacter*, resemble the patterns observed in several other significant microbial genera, including *Roseiflexus*, *Tsuneonella*, and *Cyclobacterium*. Both low and high abundances of these genera imply decreased diversity and richness of gut bacteria, which can be further associated with less weight loss outcomes (Zhang *et al.* 2009). While higher abundances of most identified genera tend to correlate with less significant weight loss after BS, increased levels of *Schaalia*, *Saccharomonospora*, and *Shimwellia* are associated with more weight loss. *Lautropia* and *Aureimonas* exhibit more complex effects that require further investigation. Although the BS surgery type is not identified as a significant biomarker contributing to the post-surgery weight reduction, HFS and HiFIT with conditional utility functions determined that RYGB and SG interact with patient age and microbial levels. More details are available in Supplementary Section S3.1.

### TCGA kidney cancer data from the KIPAN cohort

We further applied the HiFIT to analyze binary outcomes using TCGA data. Though TCGA has a large collection of publicly available clinical and omics data (Cancer Genome Atlas Research Network *et al.* 2013), we focus on the KIPAN (n=941) in our analysis. We aim to predict the patients’ survival status using normalized counts of RNA sequence data from Illumina HiSeq platform at gene level. For simplicity, we categorize patients into two groups: long-term survival (≥ 5 years of survival) and short-term survival (<5 years of survival). Out of the total 941 samples in this cohort, 193 participants achieved long-term survival, while 242 participants achieved short-term survival. The remaining samples were lost to follow-up and removed from the analysis. Additionally, expression profiles from 20 189 genes and five clinical features—age, tumor stage, histological type (cancer type), tumor purity, gender, and race—are available for these patients. Due to the large number of features and the limited sample size in the cohort, RF, SVM, and DNN fail to converge when using the full set of features, making reliable predictions impossible. Instead, we implement these algorithms using the top 1000 genes with largest variance as training input. Figure 5 presents the comparison of prediction accuracy and area under the curve (AUC) using HFS/HiFIT selected genes. HFS improves the performance of all four machine learning models and Lasso. Specifically, S-RF yields the highest prediction accuracy and AUC among all methods. Although HiFIT does not further improve the performance of machine learning algorithms, the prediction accuracy and AUC of HF-RF, HF-SVM and HF-DNN are still comparable to S-RF, S-SVM, and S-DNN, outperforming models using all top 1000 over-dispersed genes as input. Despite the minor improvement in prediction accuracy, HiFIT offers feature importance evaluation with solid statistical inference, offering in-depth understanding of disease mechanisms.

The feature-wise *P*-value for kidney cancer-associated features is summarized in Fig. 6b. We next summarize important features that are identified by at least three models from HF-DNN, HF-RF, HF-SVM, and HF-XGB. First, age and tumor stage are two important demographic features. Second, we uncover the following important genes: *KIF18A*, *PABPC1*, and *SPIC*, which are consistent with previous cancer studies. Specifically, *KIF18A* is required for chromosomally unstable tumor cells for proliferation (Marquis *et al.* 2021) and exhibits association with pan cancer survival across multiple cohorts (Liu *et al.* 2023). *PABPC1* was shown to promote cell proliferation and metastasis in pancreatic cancer (Yao *et al.* 2023). The upregulation of *SNHG4* is associated with lymph node involvement, distant metastasis, and reduced overall survival for renal cell carcinoma patients (Wu *et al.* 2020). Lastly, *SPIC* has been observed to drive cancer progression in mice (Ranzani *et al.* 2013). More details of the features can be found in Supplementary Section S3.2.

## Discussion

High-dimensional omics profiles, when combined with low-dimensional biological and clinical features, play a significant role in influencing the onset and severity of many complex human diseases. To gain fundamental insights into disease mechanisms and improve early diagnosis and precision medicine, it is essential to integrate critical molecular biomarkers with clinical risk factors. However, the high-dimensional nature of omics data, along with the intricate associations between disease outcomes and these profiles, poses substantial analytical challenges. To address these challenges, HiFIT first screens for important features using HFS, followed by refining this feature list through machine learning models.

Our research demonstrates the superior performance of HiFIT through extensive numerical simulations and two real-world data applications. In simulation studies, by combining metrics, HFS exhibits a “minimax” property, showing superior power in detecting both linear and nonlinear associations. In contrast, using a single nonlinear dependency measure may overlook biomarkers that exhibit simple correlations with disease outcomes. Although HiFIT was not specifically designed for DE or DA analysis, its success in boosting the recall and precision for nonlinear biomarker detection suggests it could have promising future applications in improving DE/DA analysis. Our real data applications include analysis of multi-omics—microbiome and genome—and their associations with various disease phenotypes. Although the biological ground truth of important genera or gene lists is unknown, the observed boost in predictive accuracy suggests that HFS and HiFIT selected highly predictive biomarkers in both studies. Remarkably, real data results highlight the practical challenges of verifying model assumptions for high-dimensional data. In the weight loss study, both simple linear and complex nonlinear effects are present (Fig. 7), while in the TCGA cohort, associations with the binary outcomes are difficult to verify through visualization. Applying parametric models for such data can easily result in model mis-specifications.

A potential direction for future research is to relax the marginal dependency assumptions when handling high-dimensional omics data. Although HFS and HiFIT demonstrated strong performance in our simulation models where feature effects are mostly additive, the marginal screening approach inherent in HFS may result in selecting highly correlated features, potentially overlooking more complex yet biologically relevant biomarkers.

In summary, the proposed HiFIT framework bridges the gap between high-dimensional omics profiles and low-dimensional biological and clinical features. By effectively handling high-dimensional data and capturing complex associations between molecular biomarkers, biological and clinical features, and disease outcomes, HiFIT facilitates robust feature importance identification. This leads to more accurate outcome predictions in high-dimensional settings, enabling scalable, interpretable, and robust disease outcome predictions. As such, HiFIT provides a valuable framework for improved disease management and personalized treatment strategies.

## Supplementary Material

btaf266_Supplementary_Data

## Data Availability

The metagenomic sequences used in this study can be found at the National Center for Biotechnology information Sequence Read Archive (https://www.ncbi.nlm.nih.gov/sra) under BiopProject PRJNA668357 and PRJNA668472. The TCGA dataset is publicly available at the LinkedOmics website (http://linkedomics.org), where the KIPAN study (i.e. KIRC, KICH, and KIRP studies) is used in our analysis.

## Code availability

HiFIT is implemented in our R package “HiFIT” which is available along with source code for replicating the simulation studies and real data applications on GitHub (https://github.com/BZou-lab/HiFIT).
