# Supplementary material for: High-dimensional biomarker identification for interpretable disease prediction via machine learning models
Source: Bioinformatics. 2025 Apr 26;41(5):btaf266. doi: 10.1093/bioinformatics/btaf266 (PMC12085223; doi:10.1093/bioinformatics/btaf266)
Supplement: btaf266_Supplementary_Data [file btaf266_supplementary_data.pdf]

Supplementary Materials of "High-dimensional Biomarker Identification for Interpretable Disease Prediction via Machine Learning Models"

Yifan Dai[1], Di Wu[1,2], Ian Carroll[3], Fei Zou[1,4*], Baiming Zou[1,5*]

1 Department of Biostatistics, University of North Carolina at Chapel Hill

2 Adams School of Dentistry, University of North Carolina at Chapel Hill

3 Department of Nutrition, University of North Carolina at Chapel Hill

4 Department of Genetics, University of North Carolina at Chapel Hill

5 School of Nursing, University of North Carolina at Chapel Hill

* Corresponding authors: fzou@bios.unc.edu, bzou@email.unc.edu

1 Method Details

1.1 Conditional Hybrid Feature Selection (HFS)

Conditional HFS is to select -omics features $\mathbf{x}_i$ associated with the disease outcomes y_i given the level of q-dimensional clinical features $\mathbf{z}_i = (z_{i1}, ..., z_{iq})^T$, i.e., to test whether $y_i \perp\!\!\!\perp \mathbf{x}_i | \mathbf{z}_i$. Conditional HFS replaces the utility functions in original HFS with their conditional counterparts. Specifically, to compute the parametric

metric, we fit the following linear or generalized linear models independently for each feature (say j^{th} feature),

$$\text{Full Model: } \mathbb{E}[y_j|x_{ij}] = g^{-1}\left(\beta_0 + \sum_{d=1}^{q} \alpha_k z_{id} + \sum_{m=1}^{M} x_{ij}^m \left(\beta_m + \sum_{d=1}^{q} \gamma_{md} z_{id}\right)\right), \tag{1}$$

$$\text{Reduced Model: } \mathbb{E}[y_j|x_{ij}] = g^{-1}\left(\beta_0 + \sum_{d=1}^{q} \alpha_d z_{id}\right), \tag{2}$$

with an appropriate link function g corresponding to the outcome type and a pre-specified order M polynomial. The adjusted R-square or McFadden's pseudo R-square, denoted as ρ_{j1}, is computed as follows:

$$\rho_{j1} = \begin{cases} 1 - \frac{n-q-1}{n-M(q+1)-q-1}\left[\frac{\sum_{i=1}^{n}(y_i - \hat{y}_i^{Full})^2}{\sum_{i=1}^{n}(y_i - \hat{y}_i^{Reduced})^2}\right], \text{ for continuous outcome,} \\ 1 - \frac{LL_{Full}}{LL_{Reduced}}, \text{ for binary outcome,} \end{cases} \tag{3}$$

where $\hat{y}_i^{Full}$ and $\hat{y}_i^{Reduced}$ are the predicted outcomes of sample i from model (1) and (2), respectively; LL_{Full} and $LL_{Reduced}$ are the respective maximal log-likelihood of model (1) and (2).

The second utility function, kernel partial correlation (KPC) coefficient, can also be extended to measure the conditional dependency [1]. Following the notation in Methods, the $n \times n$ sample kernel matrices of $(x_{ij}, \mathbf{z}_i)^T$ for each feature are defined as:

$$(K'_{\mathbf{j}})_{kl} = k\left((x_{kj}, \mathbf{z}_k)^T, (x_{lj}, \mathbf{z}_l)^T\right), \ (K_{\mathbf{z}})_{kl} = k(\mathbf{z}_k, \mathbf{z}_l).$$

The centered kernel matrices are denoted as $\tilde{K'_{\mathbf{j}}} = HK'_{\mathbf{j}}H$ and $\tilde{K_{\mathbf{z}}} = HK_{\mathbf{z}}H$. The empirical KPC between y and the jth feature conditional on $\mathbf{z}$ is defined as:

$$\hat{\rho}_{j2} = \frac{\text{tr}\left((O_{\mathbf{z}} - O'_{\mathbf{j}})^T \tilde{K}_y (O_{\mathbf{z}} - O'_{\mathbf{j}})\right)}{\text{tr}\left((I - O_{\mathbf{z}})^T \tilde{K}_y (I - O_{\mathbf{z}})\right)}, \tag{4}$$

where $O_{\mathbf{z}} = \tilde{K_{\mathbf{z}}}(\tilde{K_{\mathbf{z}}} + n\delta I)^{-1}$ and $O'_{\mathbf{j}} = \tilde{K'_{\mathbf{j}}}(\tilde{K'_{\mathbf{j}}} + n\delta I)^{-1}$ with δ being a positive constant. In our analysis, we use the Gaussian kernel $k(a, b) = \exp\{\|a - b\|_2^2/2\}$.

1.2 Bias Control and Cross-validation of HiFIT

To avoid potential biases from the selection of τ and model overfitting, HiFIT employs a nested K-fold cross-validations. First, it splits the training samples into K folds, denoted as $\{\mathcal{D}_1, ..., \mathcal{D}_K\}$. One of them, say $\mathcal{D}_1$, will be selected for validation and tuning, while predictive models and HFS scores will be built and estimated with the remaining sets. The validation fold $\mathcal{D}_1$ will be further split into two subsets, $\mathcal{D}_{1,1}$ and $\mathcal{D}_{1,2}$, where $\mathcal{D}_{1,1}$ will be first used to tune τ, while the final feature importance score will be estimated with $\mathcal{D}_{1,2}$. Then we repeat the last two steps by switching the roles of $\mathcal{D}_{1,1}$ and $\mathcal{D}_{1,2}$. After repeating the above procedure K times, we estimate the mean and variance of the null distribution of the importance score of each feature and based on which we formally test the importance of each feature as done in [2]. Of note, different features from each cross data may be selected, and HiFIT merges all selected features into a final feature set.

1.3 Setup for Simulation Studies and Real Data Analysis

In the simulation study, to compare the performance of HFS and other screening methods (Lasso, MIC, PC, SPC, and HSIC), we randomly split the data into a training set and a validation set at a ratio of 9:1. HFS is first implemented on the training set, and then the cutoff is determined on the validation set. The other methods are implemented on the full dataset, with the number of screened features being the same as HFS. In the simulation study and real data analysis sections, to compare HiFIT predictive models with corresponding baselines, we randomly split the data into a training set and a testing set at a ratio of 9:1. SVM, RF, XGB, and DNN are trained on the full training set, while S-SVM, S-RF, S-XGB, and S-DNN further use 1/9 of the training set to fine-tune the HFS cutoff. HF-SVM, HF-RF, HF-XGB, and HF-DNN conduct the 5-fold cross-validation on the training set as described above, with 100 permutations and the p-value cutoff set to 0.1. Lasso is implemented using the R package "glmnet" with 10-fold cross-validation to select its hyperparameter, while S-Lasso performed HFS with cutoff fixed at 0.6. The performance of the above predictive models is evaluated on the testing set. The above procedures are repeated for 100 times. The feature importance scores and p-values of two real datasets are computed by HiFIT on the full dataset with 5-fold cross-validation.

2 Additional Simulation Results

2.1 Oracle Models and Type-I Error Control

Supplementary Table 1 presents the performance of oracle models (defined as the models including 10 important features only as input features) and HiFIT models. The prediction accuracy of HiFIT models is close to that of the oracle models.

Supplementary Table 2 presents the Type-I error rate of HiFIT and PermFIT in simulation at the significance level of 0.05 and 0.1. Generally, HiFIT controls the Type-I error at the desired level.

Supplementary Table 1: **Prediction Performance of HiFIT Models for Coninuous Outcomes.**

		p=10*		p=500		p=1000		p=10000	
Setting	Model	MSE	PCC	MSE	PCC	MSE	PCC	MSE	PCC
Linear									
	XGBoost	4.70	0.85	4.84	0.84	4.87	0.85	5.51	0.82
	RF	5.54	0.90	5.89	0.89	6.21	0.89	7.82	0.85
	SVM	1.03	0.97	1.21	0.96	1.14	0.96	1.28	0.96
	DNN	1.12	0.96	1.30	0.96	1.27	0.96	1.43	0.95
Nonlinear									
	XGBoost	9.41	0.81	10.57	0.77	10.78	0.77	12.33	0.72
	RF	12.94	0.79	13.87	0.76	14.58	0.74	15.03	0.70
	SVM	9.25	0.80	11.42	0.74	12.94	0.69	13.98	0.66
	DNN	5.01	0.91	6.78	0.87	7.41	0.85	10.38	0.77

$*$ Oracle model including 10 important features only as input features

2.2 Simulations for Non-Gaussian Distributions

To investigate the performance of HFS and HiFIT under non-Gaussian distributions, we generate $\mathbf{x}$ from the below distributions:

- **Multinomial Distribution:** The data followed a Multinomial distribution (n_{size}, π_p), where n_{size} is the size parameter and π_p is the probability to be in each variable. We set $n_{size} = 5 \times \pi_p$, and set π_p to follow sigmoid$(\mathcal{N}(\mathbf{0}_d, \mathbf{I}_{d\times d}))$ at the beginning of each simulation.

- **Poisson Log-Normal Distribution:** The data followed a Poisson log-normal $(\mu_p, \sigma^2\Sigma_{p\times p})$, where $\Sigma_{p\times p} = \text{Diag}(\Sigma^{(1)}_{50\times50}, ..., \Sigma^{(p/50)}_{50\times50})$ is block diagonal, with the diagonal elements of each block $\Sigma^{(i)}_{50\times50}$ to be 1 and off-diagonal elements to be 0.1. The mean vector μ_p was sampled from $\mathcal{N}(0, \mathbf{I}_p)$ at the

Supplementary Table 2: **Type I Error of HiFIT and PermFIT at the significance level of α in Simulation.**

Setting	Model	$p = 500$		$p = 1000$		$p = 10000$	
		$\alpha = 0.05$	$\alpha = 0.10$	$\alpha = 0.05$	$\alpha = 0.10$	$\alpha = 0.05$	$\alpha = 0.10$
Linear	HF-DNN	0.09	0.11	0.06	0.08	0.08	0.16
	PermFIT-DNN	0.13	0.20	0.11	0.16	–	–
	HF-RF	0.06	0.09	0.05	0.06	0.11	0.16
	PermFIT-RF	0.08	0.14	0.06	0.13	–	–
	HF-SVM	0.08	0.12	0.01	0.10	0.07	0.13
	PermFIT-SVM	0.12	0.21	0.10	0.19	–	–
	HF-XGB	0.06	0.09	0.03	0.12	0.08	0.14
	PermFIT-XGB	0.09	0.16	0.03	0.07	–	–
Nonlinear	HF-DNN	0.04	0.07	0.05	0.07	0.07	0.13
	PermFIT-DNN	0.04	0.10	0.04	0.05	–	–
	HF-RF	0.04	0.09	0.04	0.08	0.06	0.10
	PermFIT-RF	0.09	0.13	0.05	0.12	–	–
	HF-SVM	0.05	0.09	0.08	0.16	0.02	0.06
	PermFIT-SVM	0.05	0.07	0.03	0.08	–	–
	HF-XGB	0.05	0.10	0.05	0.10	0.05	0.09
	PermFIT-XGB	0.09	0.14	0.06	0.10	–	–

beginning of each simulation and fixed when sampling all 500 observations.

These two distributions are commonly used to model correlated and over-dispersed count data. The other settings are the same for the Gaussian scenarios. Supplementary Figures 1 $\sim$ 6 present the results. As expected, HFS and HiFIT are robust to different underlying distributions.

2.3 Simulations for a Mixture of Linear and Nonlinear Effects

To investigate the performance of HiFIT under more realistic settings, we consider the following model

$$y \sim \sum_{j=1}^{4} \beta_j x_j - \sum_{j=5}^{8} 2\log(2x_j^2 + 1) + x_9 \exp(x_{10}) + \epsilon,$$

where $\beta_j \sim \mathcal{U}(0.5, 4.5)$. In this setting, both linear and nonlinear effects are included, with other data structures the same as the linear or nonlinear scenarios. Supplementary Figures 7 $\sim$ 9 present the results that are similar to the nonlinear settings.

2.4 Simulations for Binary Outcomes

We also conducted simulation studies on binary data generated from the following models, i.e., one for generalized linear impacts and the other for generalized nonlinear effects.

$$
\begin{aligned}
\text{logit}[P(y|\mathbf{x})] &= \sum_{j=1}^{10} \beta_j x_j \\
\text{logit}[P(y|\mathbf{x})] &= \sum_{j=1}^{4} 4\sin(2x_j) - \sum_{j=5}^{8} 4\log(2x_j^2 + 1) + 2x_9 \exp(x_{10}) + 13
\end{aligned}
$$

where $\beta_j \sim \mathcal{U}(2, 3)$. All other data structures are generated in the same way as in the continuous scenarios. Results are presented in Supplementary Figures 10 $\sim$ 13. Similar to the results for continuous outcomes, HFS effectively selects most important features, while HiFIT further reduces noise features, thereby enhancing the prediction accuracy of machine learning models.

3 Details of Real Data Applications

In this section, we present additional results to offer further insights into the biomarkers identified by HFS and HiFIT in two real-world datasets: a microbiome-weight loss study following bariatric surgery and a kidney pan-cancer cohort from The Cancer Genome Atlas (TCGA).

3.1 Microbiome-based Analysis of Weight Loss after Bariatric Surgery (BS)

Supplementary Figure 14 shows the t-distributed Stochastic Neighborhood Embedding (t-SNE, [3]) visualizations of all microbial genera, HFS-identified genera, and HiFIT-identified genera. Despite the relatively weak microbial signal, the embeddings of HFS and HiFIT genera more clearly separate patient groups with different levels of weight loss.

Supplementary Figure 15 illustrates the abundance of HFS genera, highlighting their differential abundance across patients with varying degrees of weight loss. Consistent with the analysis in the Results and Discussion section, intermediate levels of most HFS microbial genera appear to be associated with better weight loss outcomes.

To explore the diversity of HFS and HiFIT genera, we performed clustering and correlation analyses. Supplementary Figure 16 presents the results of the hierarchical clustering significance analysis [4] for both HFS and HiFIT genera, identifying three major genera clusters that encompass approximately half of the HFS-identified genera. Notably, the two most important genera—*Hyphobacterium* and *Panacibacter*—belong to distinct clusters. Another significant genus, *Schaalia*, comes from a separate cluster with distinct patterns from the others. Despite the presence of highly correlated clusters, the results also reveal at least six smaller clusters with distinct abundance patterns, suggesting diversity among the HFS genera.

Supplementary Figure 17 illustrates the correlations among HFS genera. While most genera show strong correlations, a few, including *Schaalia*, are less correlated with the others. Further investigation into these genera may provide valuable insights into the role of the fecal microbiome in post-surgery weight loss.

To investigate the interactive effects of fecal microbial abundance and BS type for personalized treatments, we conducted HiFIT using the conditional utility functions (3) and (4), setting $\mathbf{z}_i$ as the BS type. Supplementary Figure 18 shows the predictive accuracy of both HFS and HiFIT models, where S-SVM, HF-SVM, S-DNN, and HF-DNN outperform their counterparts in Figure 5. The superior performance of these models implies that post-surgery fecal microbiome levels may interact with the type of BS received. Supplementary Figure 19 further illustrates HiFIT-identified features interacting with surgery type. Roux-en-Y Gastric Bypass (RYGB) appears to achieve better weight loss outcomes than Sleeve Gastrectomy (SG) in patients aged 40–50. Additionally, RYGB demonstrates significantly better outcomes for patients with extreme levels of *Silicimonas, Sulfuracidifex, Miniimonas, Streptococcus, Flavobacterium*, and *Lancefieldella.* This consistently better outcome in RYGB patients may indicate a more diverse and balanced gut microbiome environment. Other microbial genera, such as *Tatumella* and *Parabacteroides*, show a more complex correlation structure, hinting at the potential for precision treatment based on microbial abundance.

3.2 Analysis of Kidney Pan Cancer Survival Time in the TCGA Cohort

Supplementary Figure 21 shows the t-SNE visualizations of the top 1,000 over-dispersed genes, HFS-identified genes, and HiFIT-identified genes. The embedding of the top 1,000 genes reveals that the genomic profiles of long-term survival patients are similar to those of short-term survival patients, suggesting that these genes

may be too noisy to capture cancer-related patterns, potentially leading to poor predictive performance in downstream analyses. In contrast, the embeddings of HFS and HiFIT genes more clearly separate patient groups with different survival times.

Supplementary Figure 22 illustrates the expression profiles of HiFIT genes, revealing their differential expression across patients with different survival times. As expected, all HiFIT genes show significant differential expression. Notably, the expression patterns highlight the heterogeneity within the short-term survival group. Specifically, genes from *POLQ* to *ZIC2* in Supplementary Figure 22 are highly expressed in certain short-term survival patients, while genes from *CCDC19* to *CCNL2* exhibit higher expression in most of the other patients. This finding underscores HiFIT's ability to identify a diverse and heterogeneous set of genes associated with disease outcomes.

To further explore the diversity of HFS and HiFIT genes, we performed clustering and correlation analyses. Supplementary Figure 23 presents the results of the hierarchical clustering significance analysis for both HFS and HiFIT genes. The analysis reveals distinct gene clusters, with some genes exhibiting similarity and potentially forming a latent group. Supplementary Figure 24 illustrates the correlation among HFS genes. While many genes are highly correlated, forming clusters, several genes –including *FLJ11235*, *LRBA*, *NR3C2*, *SLC25A4*, and *SLC4A5* – are less correlated with other selected genes. They may provide unique insight to the disease mechanism.

HiFIT can also provide insights to the interactive effects of histological type and molecular traits. As shown in Supplementary Figure 18, the improvement of HFS and HiFIT for survival prediction is similar to that in Figure 5. Supplementary Figure 25 exhibits no significant interaction effects of histological type and gene expression on patients' survival time.

References

[1] Zhen Huang, Nabarun Deb, and Bodhisattva Sen. Kernel partial correlation coefficient — a measure of conditional dependence. Journal of Machine Learning Research, 23(216):1–58, 2022.

[2] Xinlei Mi, Baiming Zou, Fei Zou, and Jianhua Hu. Permutation-based identification of important

biomarkers for complex diseases via machine learning models. Nature communications, 12:3008, 2021.

[3] Laurens Van der Maaten and Geoffrey Hinton. Visualizing data using t-sne. Journal of machine learning research, 9(11), 2008.

[4] Patrick K Kimes, Yufeng Liu, David Neil Hayes, and James Stephen Marron. Statistical significance for hierarchical clustering. Biometrics, 73(3):811–821, 2017.

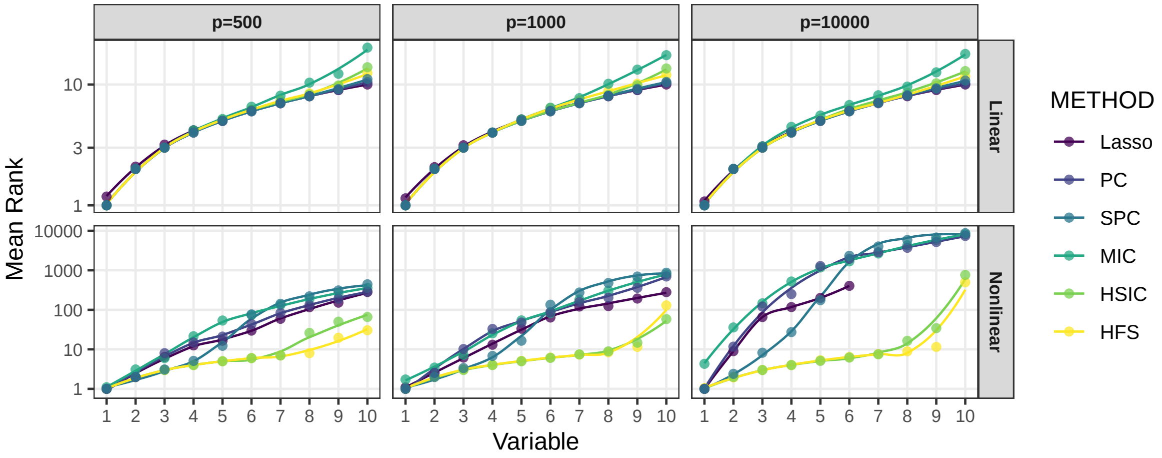

Supplementary Figure 1: **Average Rank of Important Features Selected by Pre-Screening Methods for Multinomial distributions.** The x-axis denotes the number of selected important features, and the corresponding value of the y-axis represents the average rank of this feature over 100 repetitions. The curves are generated by locally polynomial smoothing.

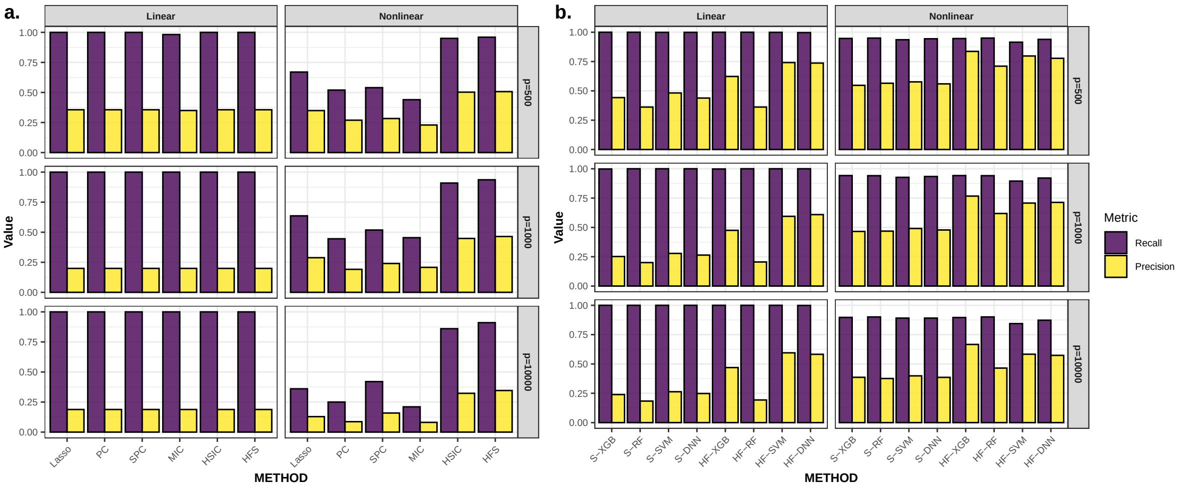

Supplementary Figure 2: **High-dimensional Feature Pre-Screening and Selection Results for Multinomial Distributions.** **(a)** Performance of feature pre-screening methods. **(b)** Feature selection results of HiFIT models. Recall and precision are averaged over 100 simulations.

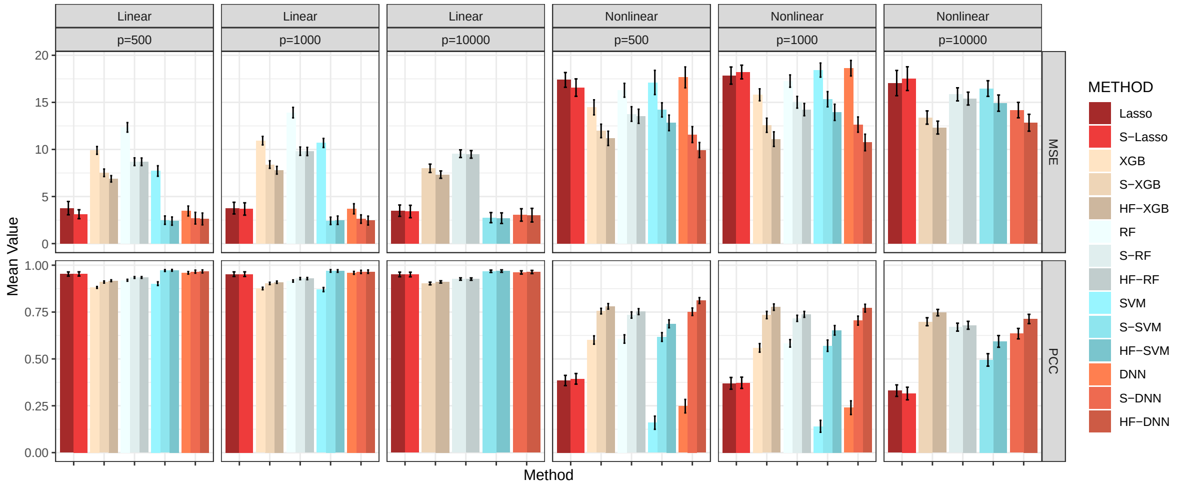

Supplementary Figure 3: **Average MSE and PCC of Models in Comparison for Multinomial Distributions.** Lasso, XGB, RF, SVM, and DNN: specific models with all features; S-Lasso, S-XGB, S-RF, S-SVM, S-DNN: specific models with HFS pre-screening; HF-XGB, HF-RF, HF-SVM, HF-DNN: specific models with HiFIT feature selection. Simulation in each scenario is repeated 100 times.

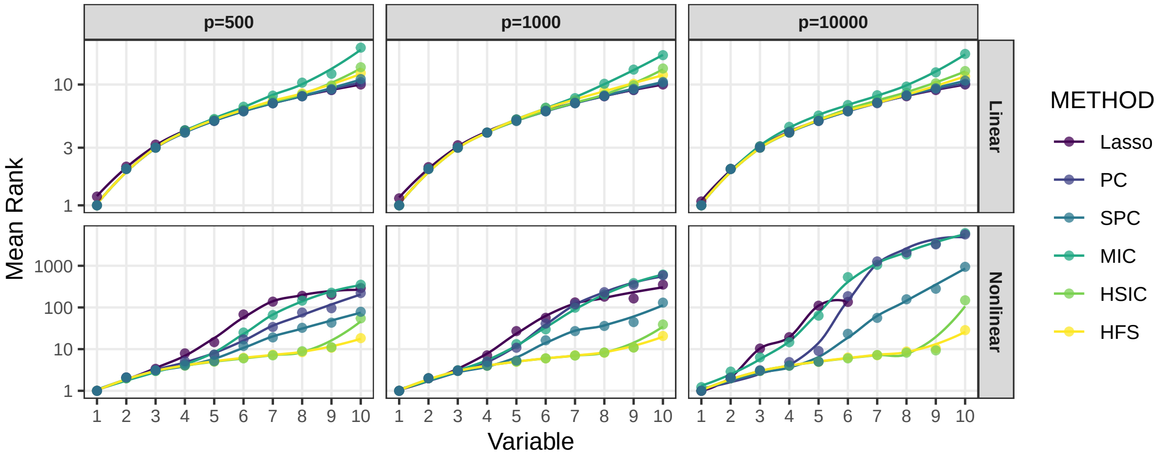

Supplementary Figure 4: **Average Rank of Important Features Selected by Pre-Screening Methods for Poisson Log-Normal Distributions.** The x-axis denotes the number of selected important features, and the corresponding value of the y-axis represents the average rank of this feature over 100 repetitions. The curves are generated by locally polynomial smoothing.

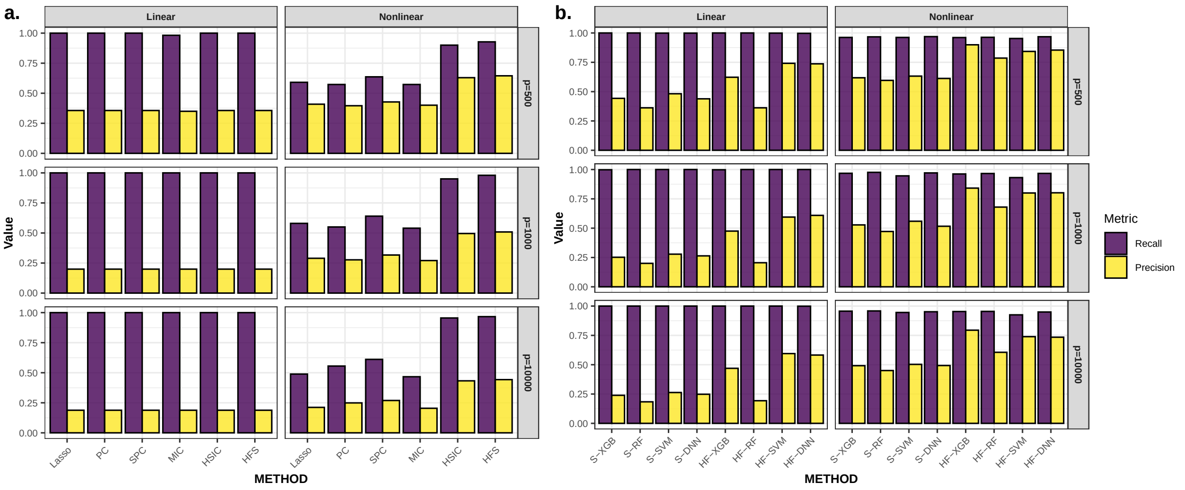

Supplementary Figure 5: **High-dimensional Feature Pre-Screening and Selection Results for Poisson Log-Normal Distributions.** **(a)** Performance of feature pre-screening methods. **(b)** Feature selection results of HiFIT models. Recall and precision are averaged over 100 simulations.

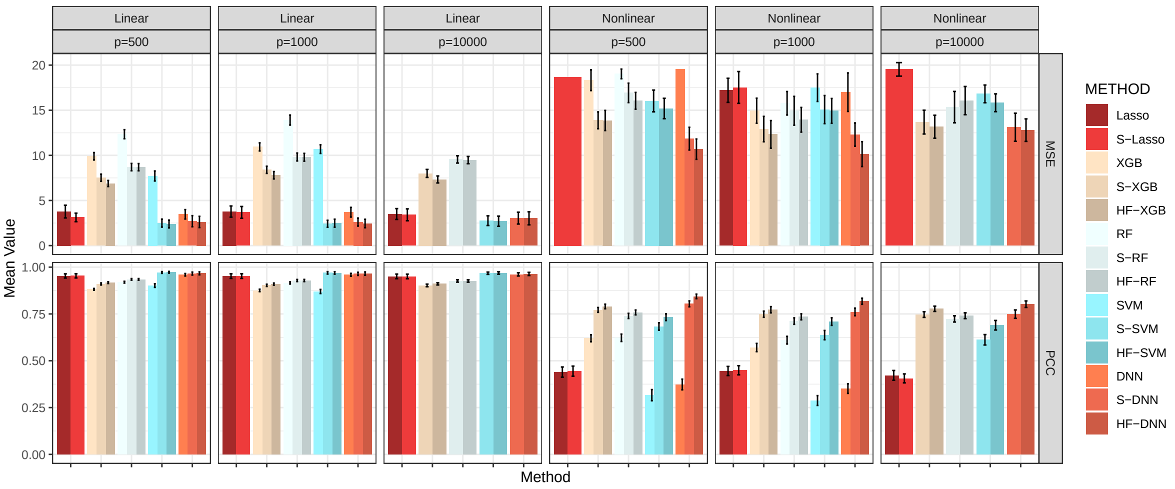

Supplementary Figure 6: **Average MSE and PCC of Models in Comparison for Poisson Log-Normal Distributions.** Lasso, XGB, RF, SVM, and DNN: specific models with all features; S-Lasso, S-XGB, S-RF, S-SVM, S-DNN: specific models with HFS pre-screening; HF-XGB, HF-RF, HF-SVM, HF-DNN: specific models with HiFIT feature selection. Simulation in each scenario is repeated 100 times.

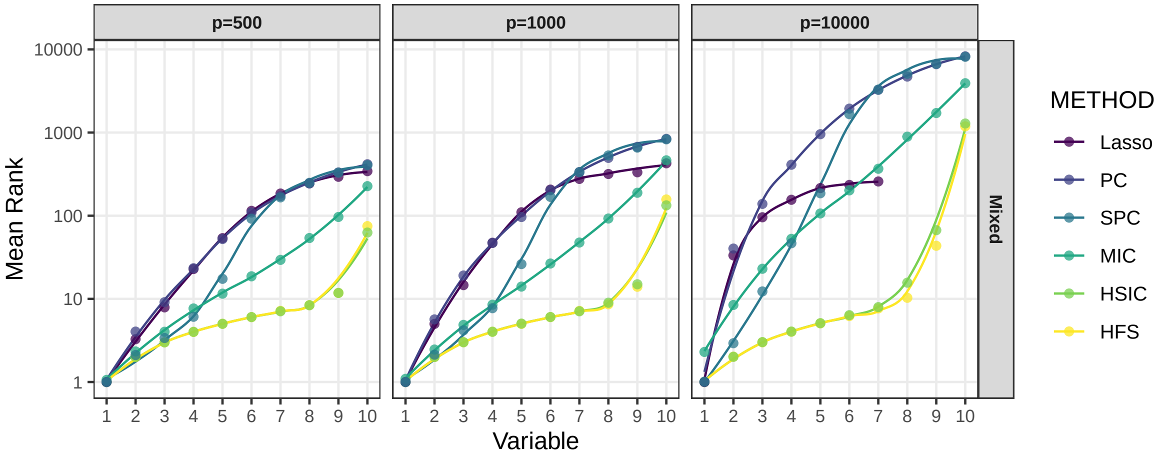

Supplementary Figure 7: **Average Rank of Important Features Selected by Pre-Screening Methods for Mixed Linear and Nonlinear Settings.** The x-axis denotes the number of selected important features, and the corresponding value of the y-axis represents the average rank of this feature over 100 repetitions. The curves are generated by locally polynomial smoothing.

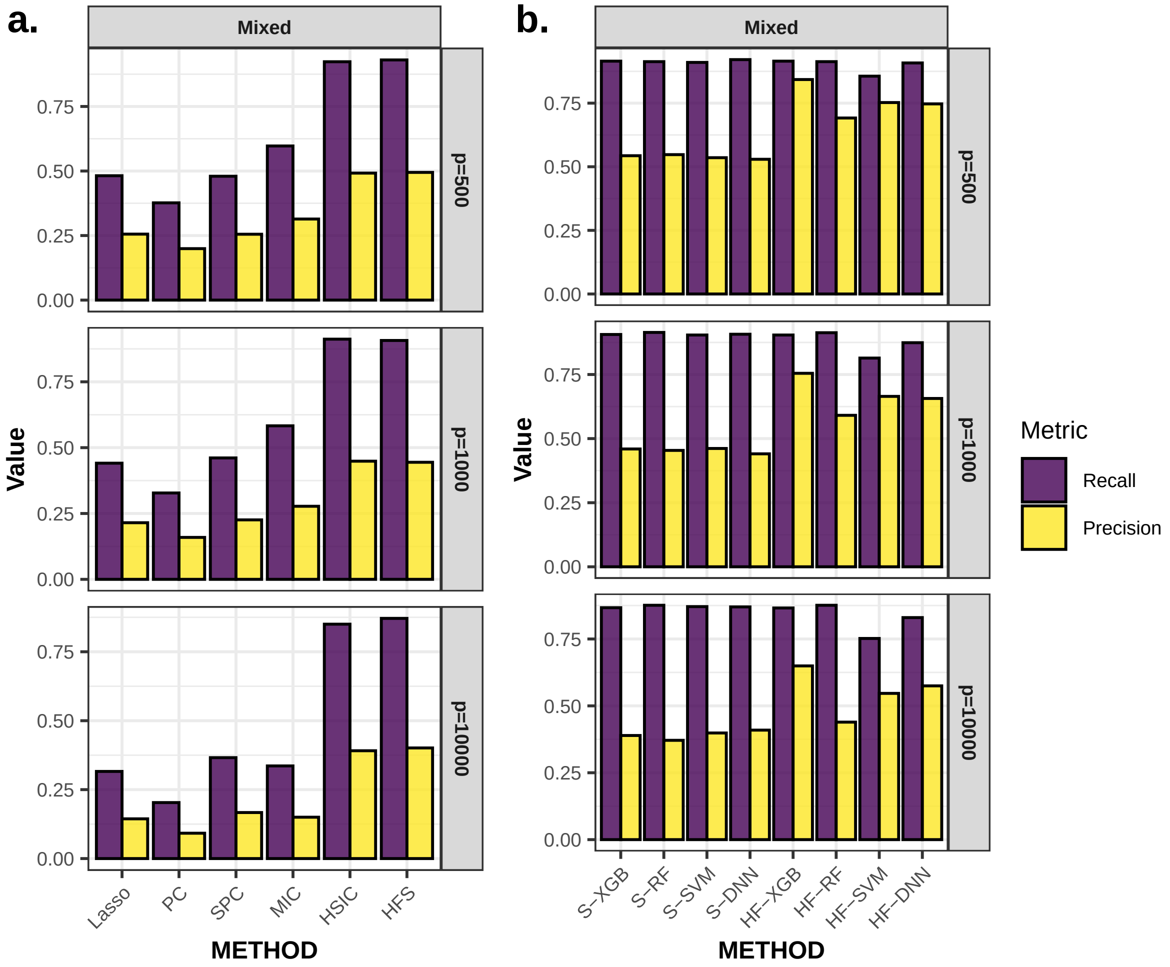

Supplementary Figure 8: **High-dimensional Feature Pre-Screening and Selection Results for Mixed Linear and Nonlinear Settings.** **(a)** Performance of feature pre-screening methods. **(b)** Feature selection results of HiFIT models. Recall and precision are averaged over 100 simulations.

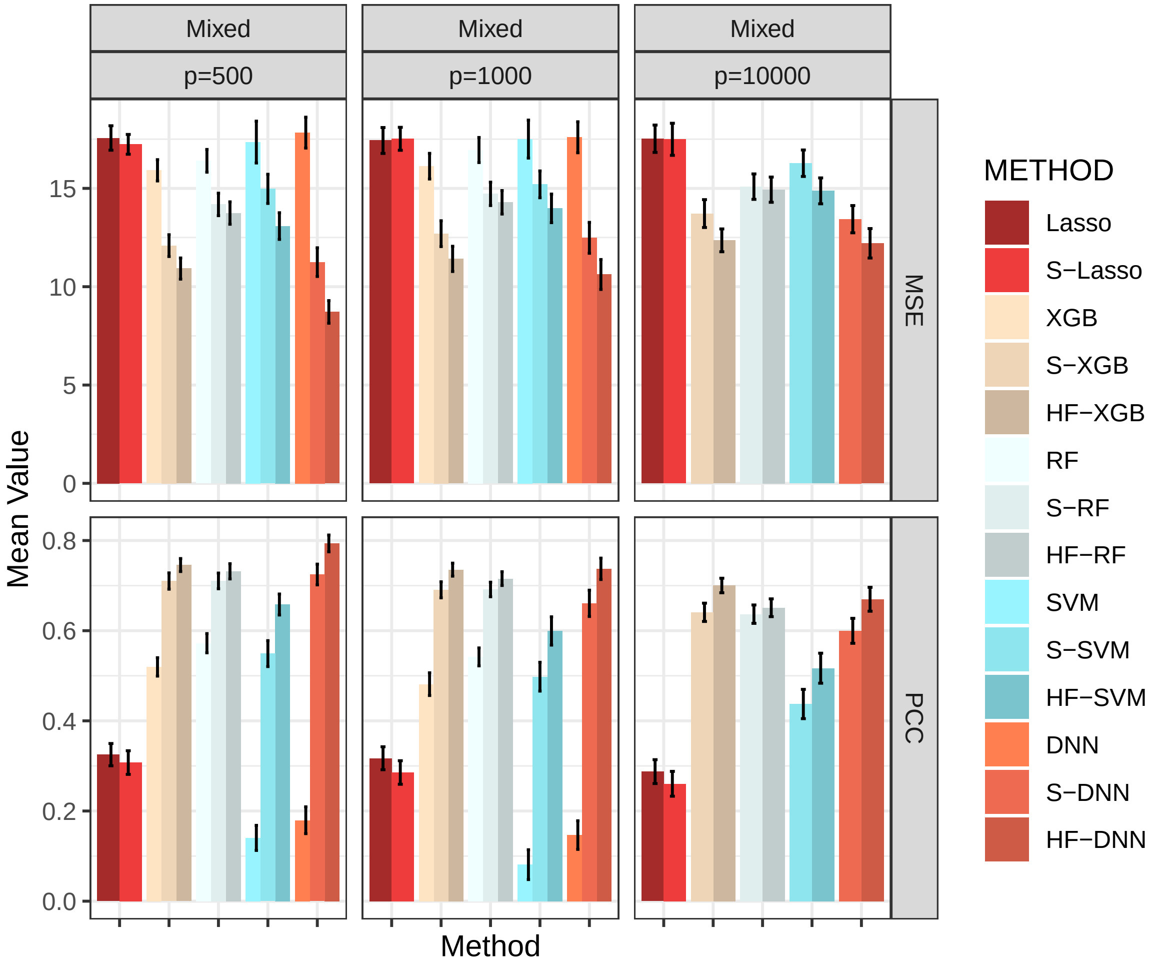

Supplementary Figure 9: **Average MSE and PCC of Models in Comparison for Mixed Linear and Nonlinear Settings.** Lasso, XGB, RF, SVM, and DNN: specific models with all features; S-Lasso, S-XGB, S-RF, S-SVM, S-DNN: specific models with HFS pre-screening; HF-XGB, HF-RF, HF-SVM, HF-DNN: specific models with HiFIT feature selection. Simulation in each scenario is repeated 100 times.

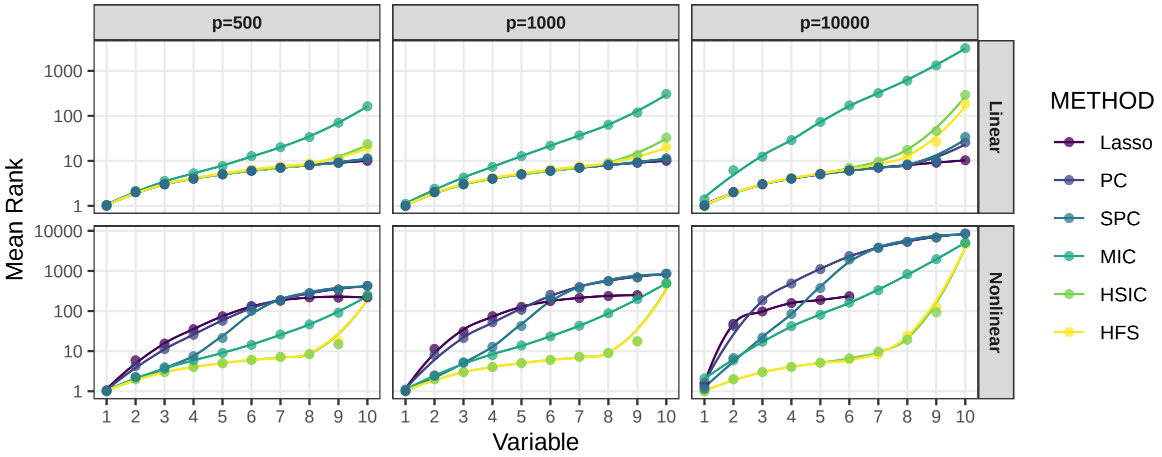

Supplementary Figure 10: **Average Rank of Important Features Selected by Pre-Screening Methods on Binary Outcomes.** The x-axis denotes the number of selected important features, and the corresponding value of the y-axis represents the average rank of this feature over 100 repetitions. The curves are generated by locally polynomial smoothing.

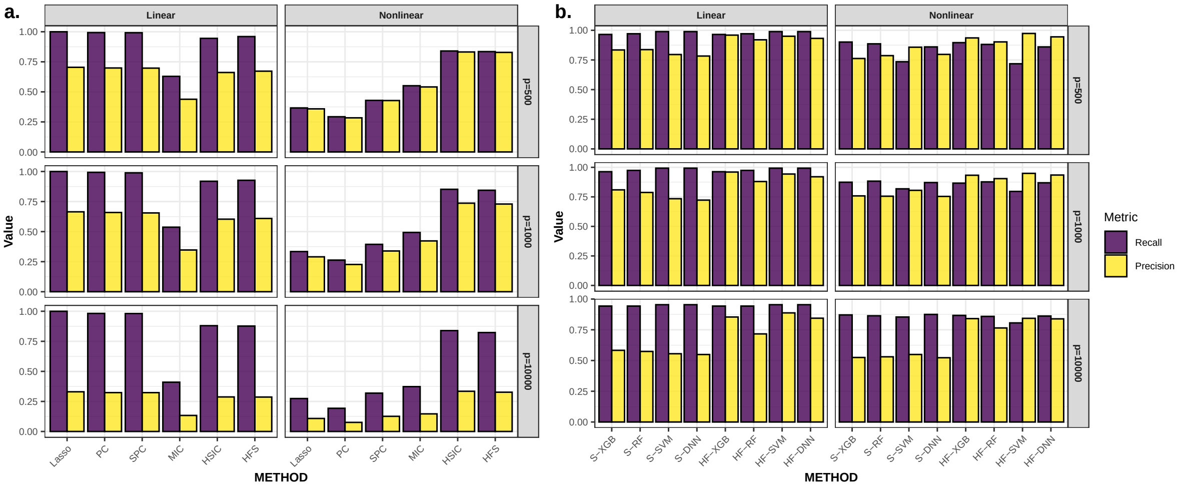

Supplementary Figure 11: **High-dimensional Feature Pre-Screening and Selection Results on Binary Outcomes.** **(a)** Performance of feature pre-screening methods. **(b)** Feature selection results of HiFIT models. Recall and precision are averaged over 100 simulations.

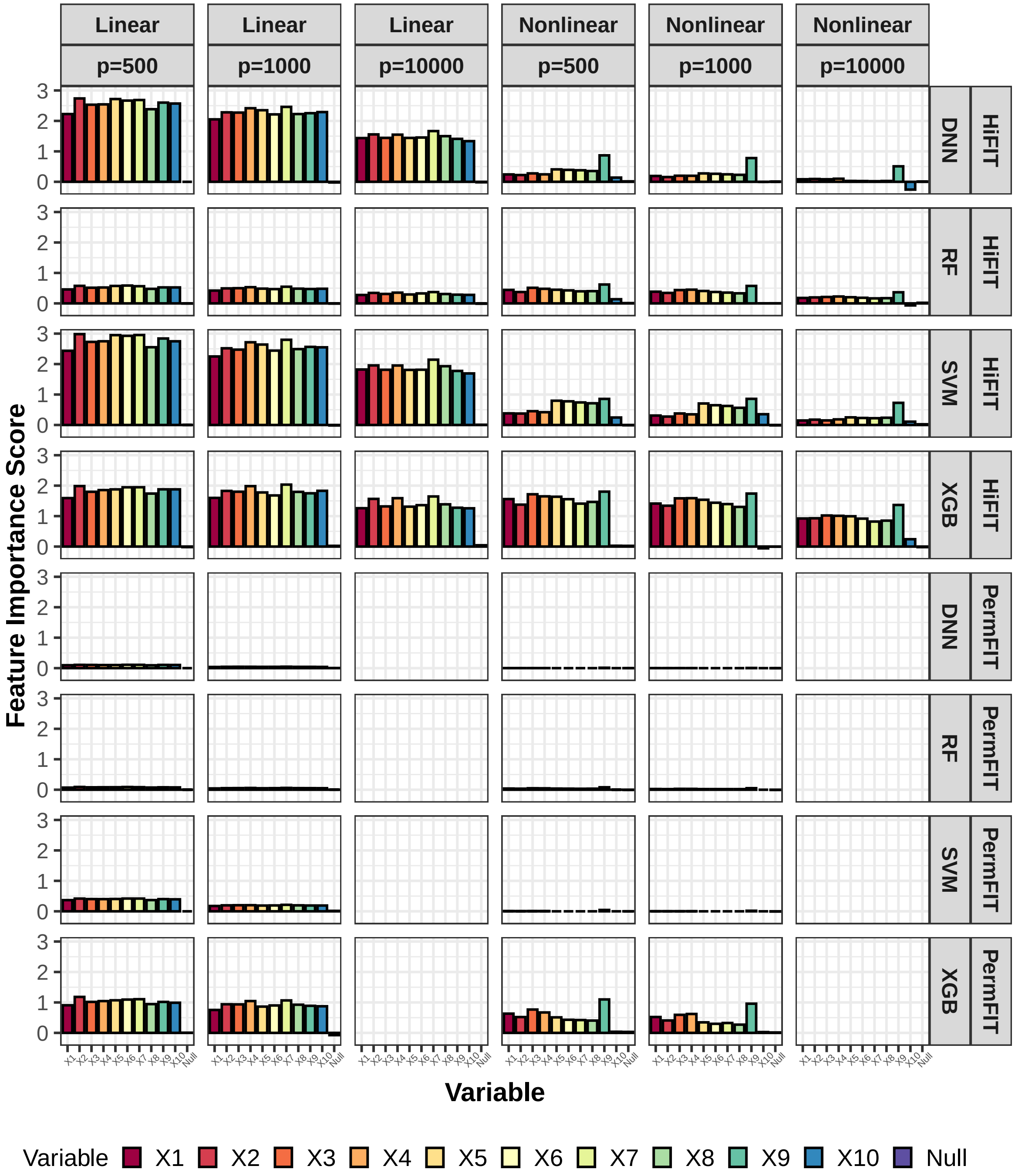

Supplementary Figure 12: **HiFIT Feature Interpretation Results on Binary Outcomes.** Average feature importance scores for 10 important variables (denoted as $X_1, ..., X_{10}$) and the feature set of nuisance features (denoted as null) over 100 repetitions. Importance scores of features not selected by HFS are set to zero.

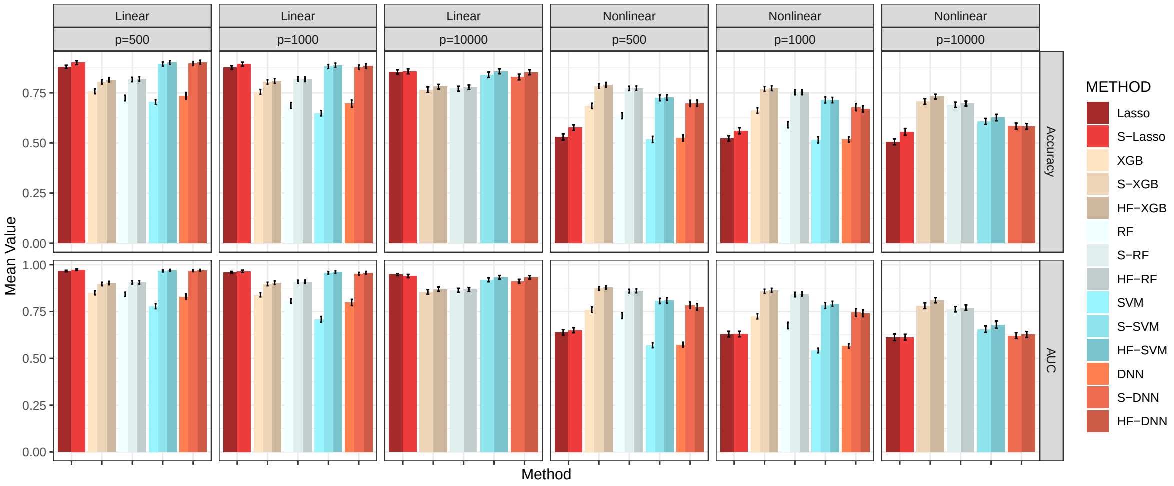

Supplementary Figure 13: **Average Accuracy and AUC for Methods in Comparison on Binary Outcomes.** Lasso, XGB, RF, SVM, and DNN: specific models with all features; S-Lasso, S-XGB, S-RF, S-SVM, S-DNN: specific models with HFS pre-screening; HF-XGB, HF-RF, HF-SVM, HF-DNN: specific models with HiFIT feature selection. Simulation in each scenario is repeated 100 times.

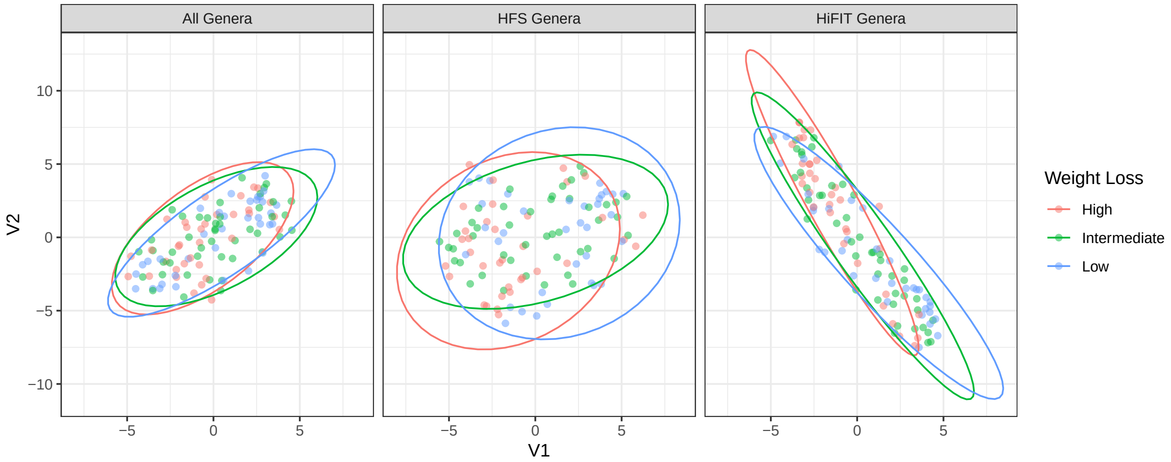

Supplementary Figure 14: **t-Distributed Stochastic Neighborhood Embedding (t-SNE) for Patients in the Weight Loss Cohort Using all Microbial Genera, HFS Genera, and HiFIT Genera.** In this analysis, patients are categorized based on their BMI reduction. Those with a BMI reduction greater than 15 are classified as having high weight loss, those with a BMI reduction between 10 and 15 as intermediate weight loss, and those with a BMI reduction of less than 10 as low weight loss.

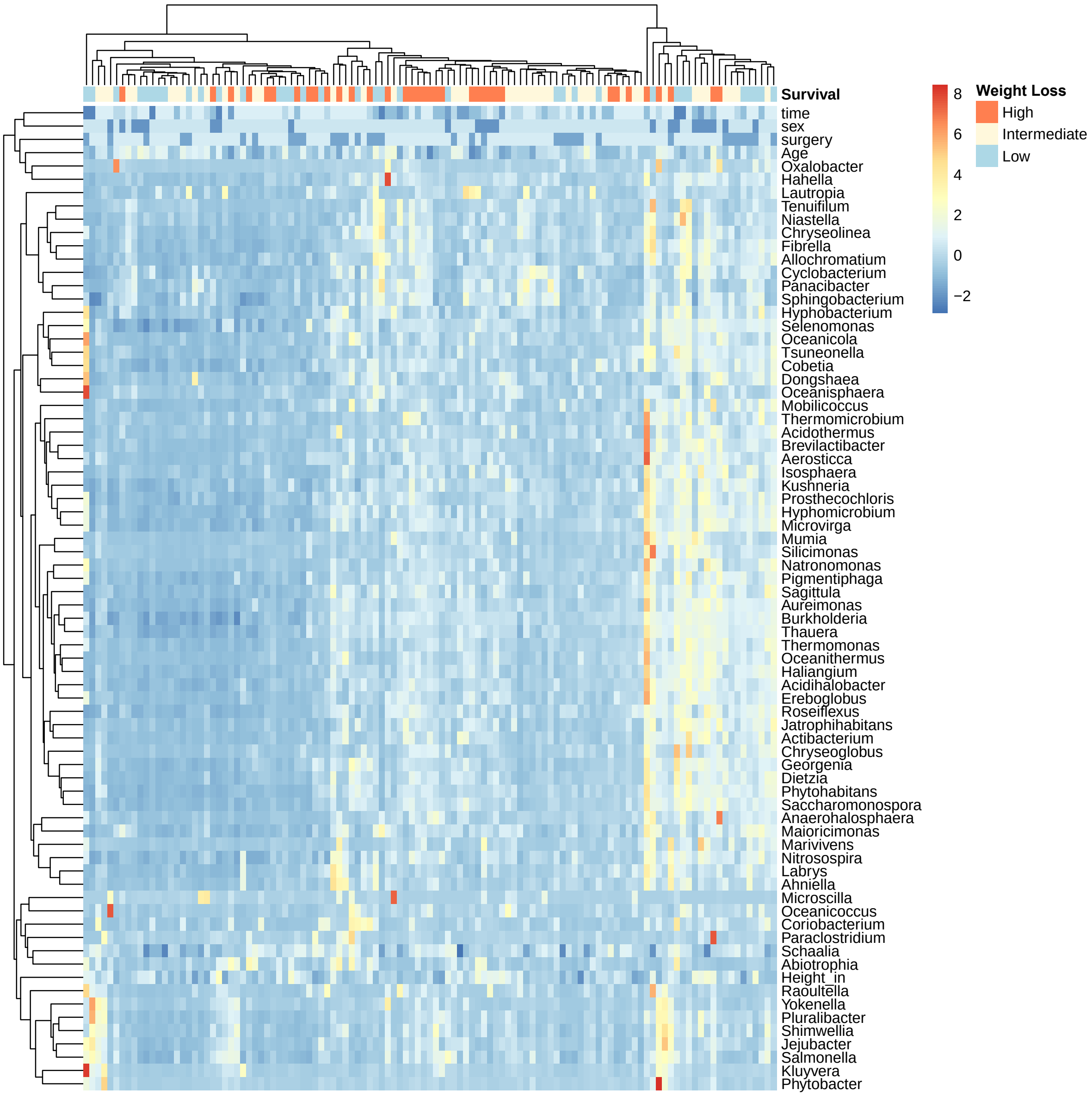

Supplementary Figure 15: **Normalized Abundance Heatmap of HFS Microbial Genera in the Weight Loss Cohort.** The dendrogram along the x-axis illustrates the hierarchical clustering of patients, while the dendrogram on the y-axis represents the hierarchical clustering of HFS Microbial Genera and Demographic Features. The color bar along the x-axis indicates the levels of post-surgery weight loss.

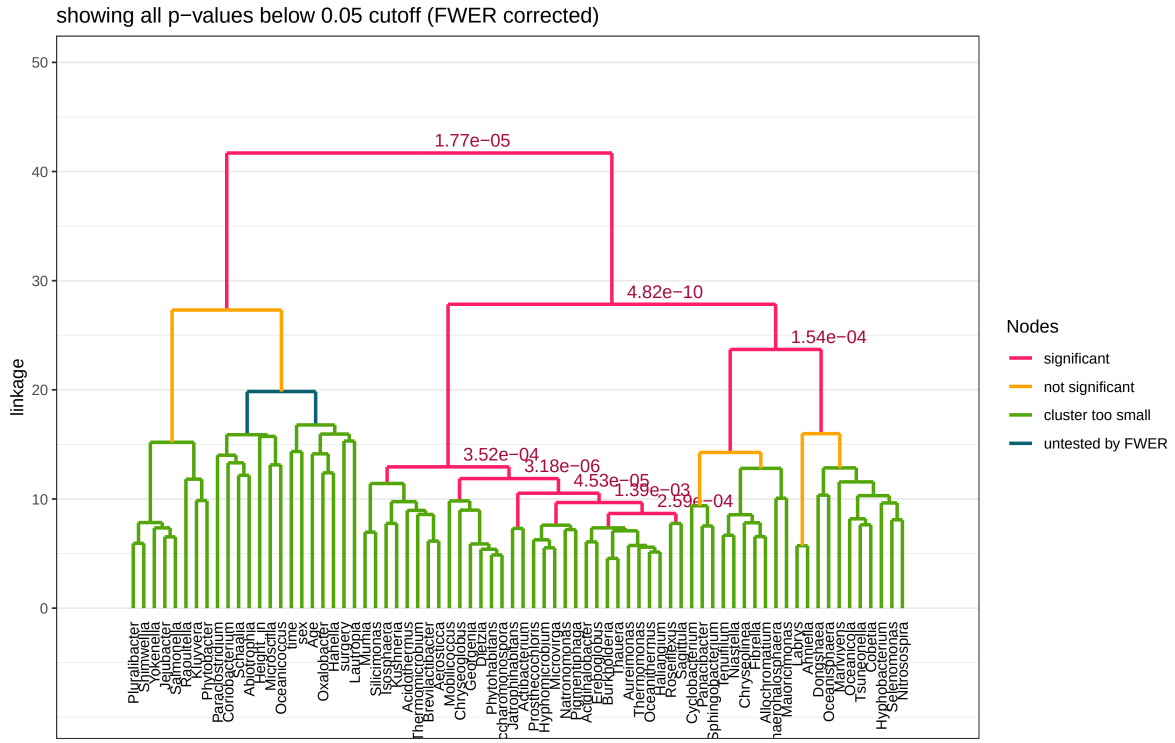

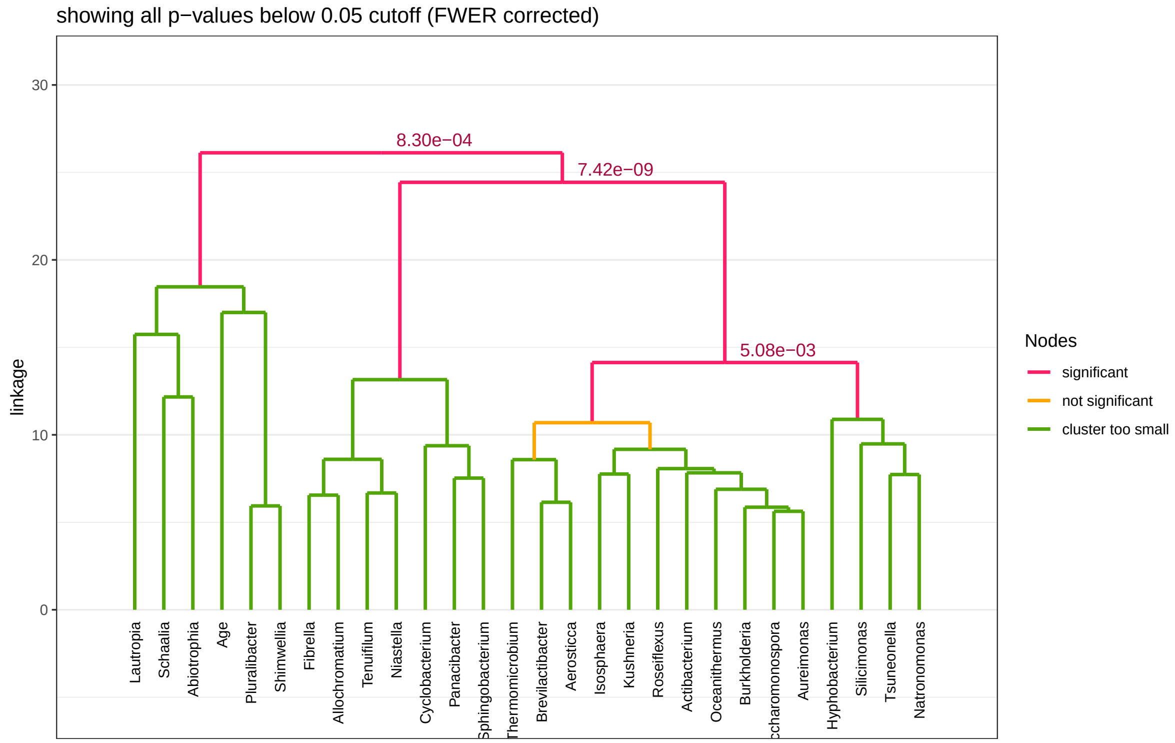

Supplementary Figure 16: **Significance Analysis of Hierarchical Clustering for HFS Microbial Genera (upper) and HiFIT Microbial Genera (lower) in the Weight Loss Cohort.** Significant nodes indicate that the genera grouped under these nodes exhibit differential abundance, whereas non-significant nodes suggest similar abundance patterns of genera across patients.

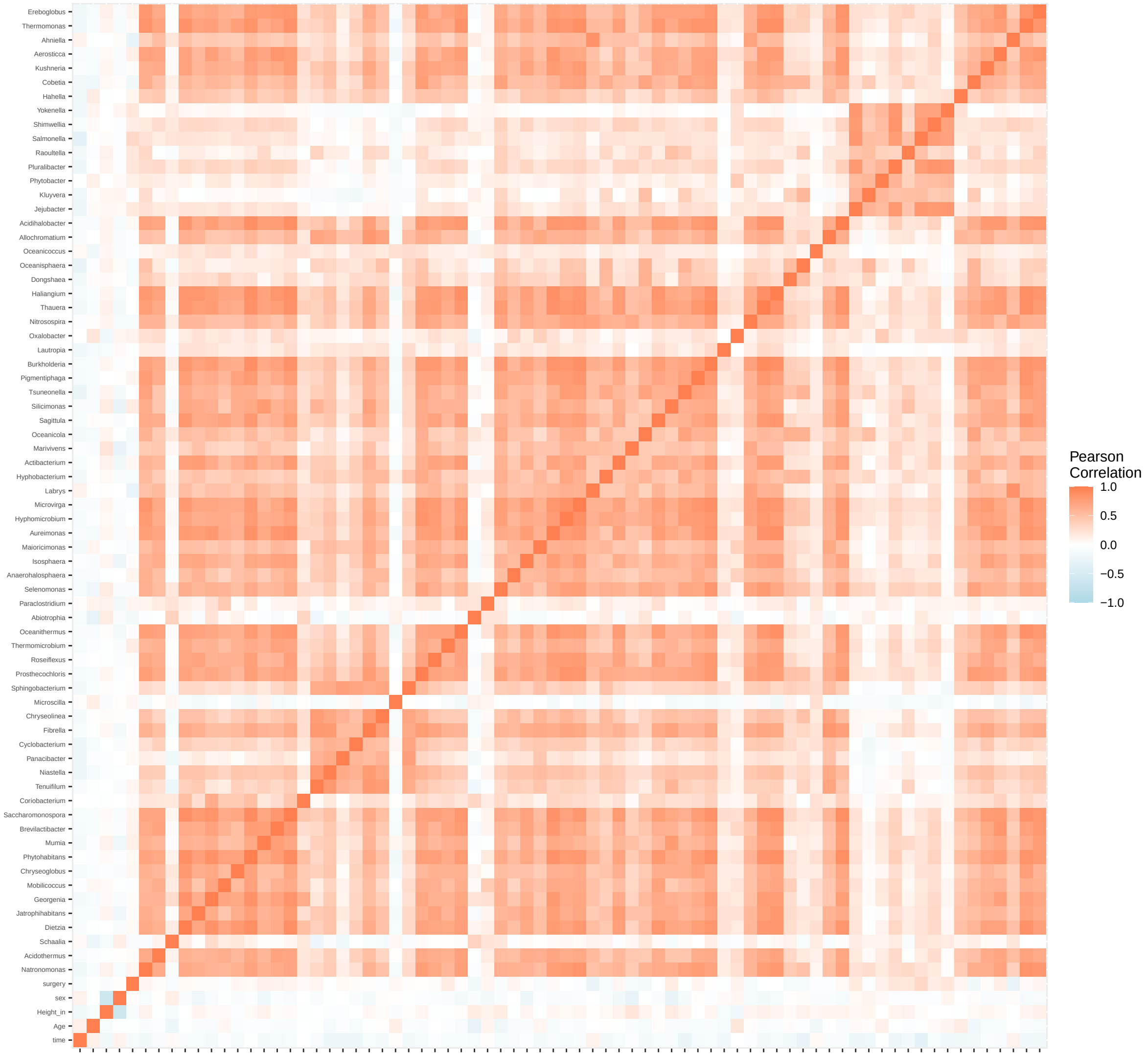

Supplementary Figure 17: **The Correlation Matrix of HFS Microbial Genera and Demographic Features in the Weight Loss Cohort.**

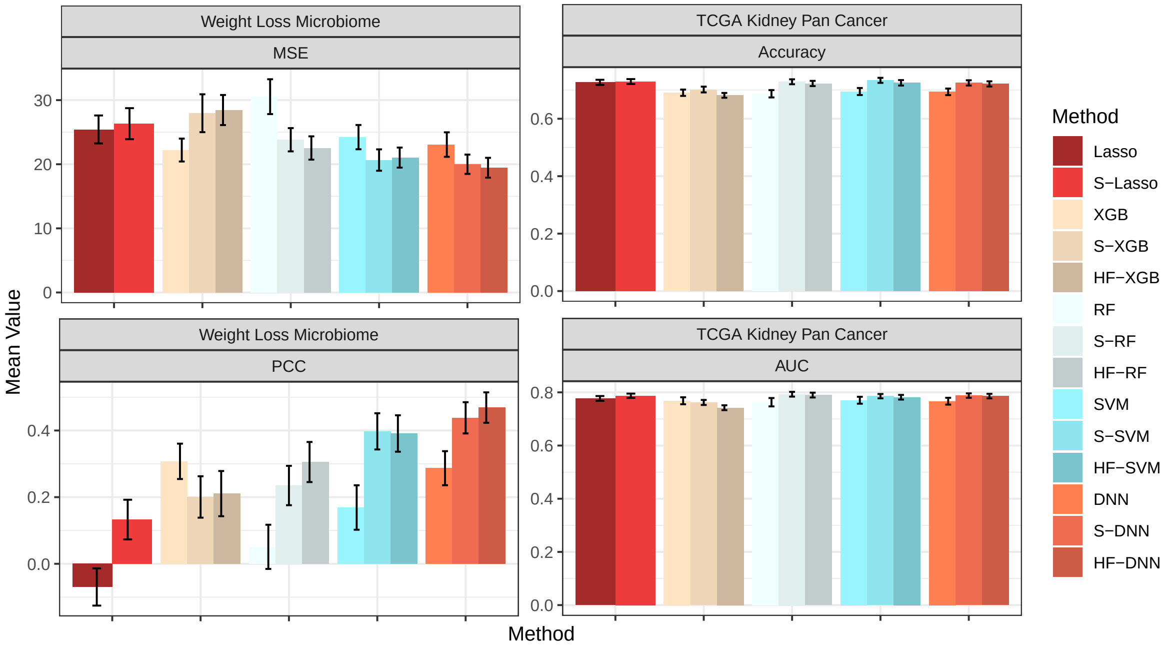

Supplementary Figure 18: **Model Performance for the Real Data Analysis using Conditional HFS Utility Functions.** The first column presents the MSE and PCC for the weight loss microbiome study. The second colume presents the accuracy and AUC for TCGA kidney pan cancer cohort. All metrics are averaged on separate testing sets consisting of 10% observations with random repeats for 100 times.

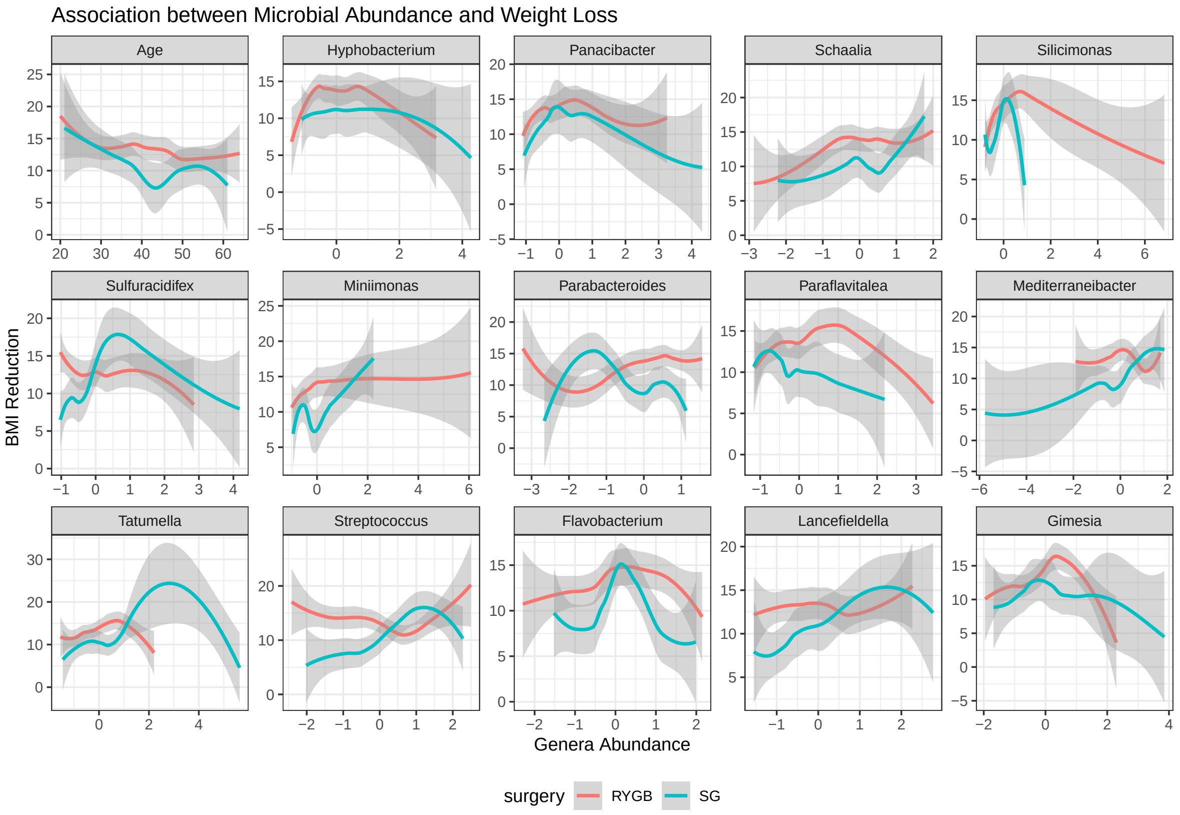

Supplementary Figure 19: **Association across BMI Reduction, Bariatric Surgery Type, and Microbiome Abundance.** The smoothing curves and 95% confidence bands are obtained by locally polynomial regression.

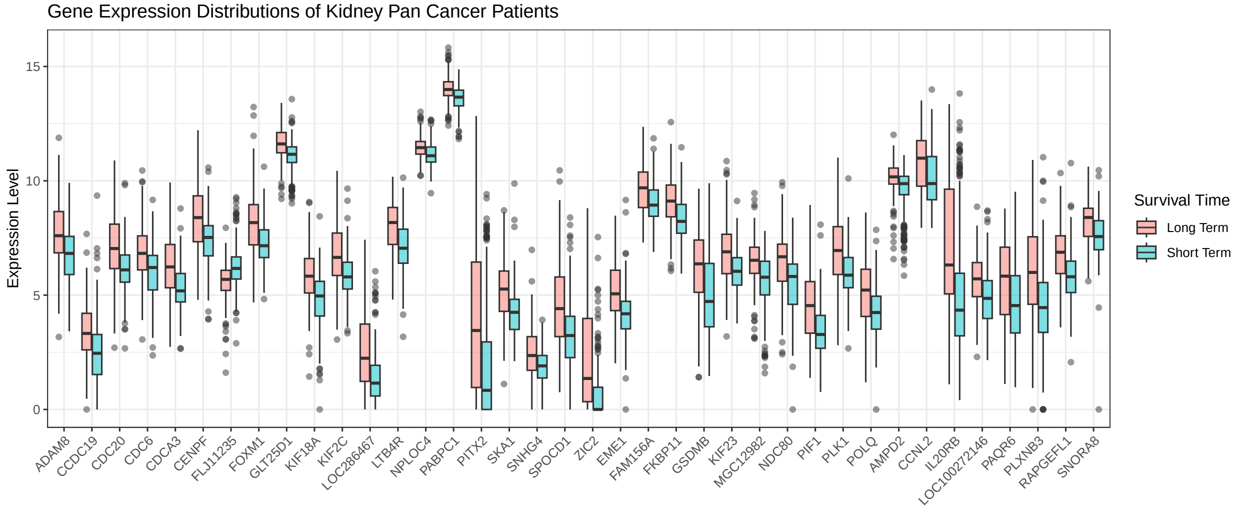

Supplementary Figure 20: **Association between Kidney Pan Cancer Survival and Gene Expression in the TCGA cohort.** The x-axis consists of important genes identified by at least one HiFIT model in Figure 6b, and the y-axis represents the logarithm gene expression.

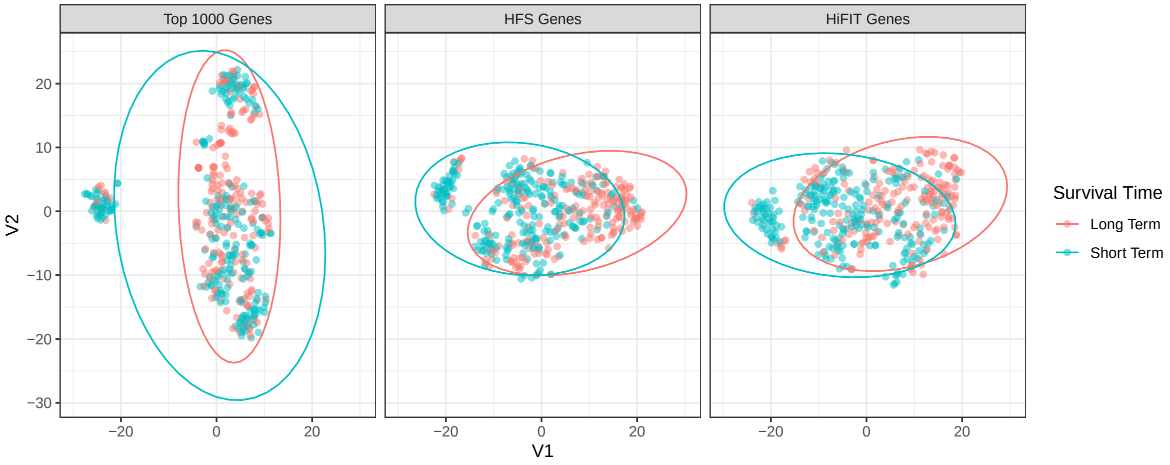

Supplementary Figure 21: **t-Distributed Stochastic Neighborhood Embedding (t-SNE) for Kidney Pan Cancer Patients in the TCGA cohort using Top 1000 Over-Dispersed Genes, HFS Genes, and HiFIT Genes.** The gene sets identified by HFS and HiFIT result in more distinguishable embeddings compared to the top 1000 over-dispersed genes. While some short-term survival patients show distinct patterns based on the top 1000 over-dispersed genes, long-term survival patients tend to overlap with certain clusters of short-term survival patients. However, the genes selected by HFS and HiFIT enable better identification of long-term survival patients from other gene expression profiles.

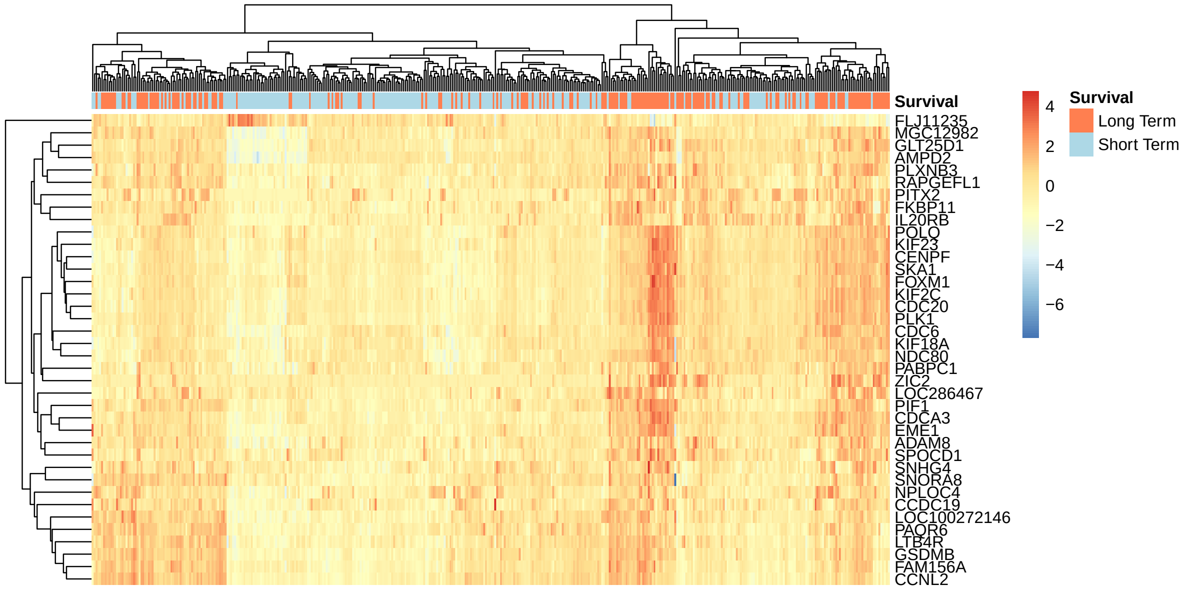

Supplementary Figure 22: **Normalized Expression Heatmap of HiFIT Genes in the TCGA Cohort.** The dendrogram along the x-axis illustrates the hierarchical clustering of patients, while the dendrogram on the y-axis represents the hierarchical clustering of HiFIT genes. The color bar along the x-axis indicates the survival times of the patients.

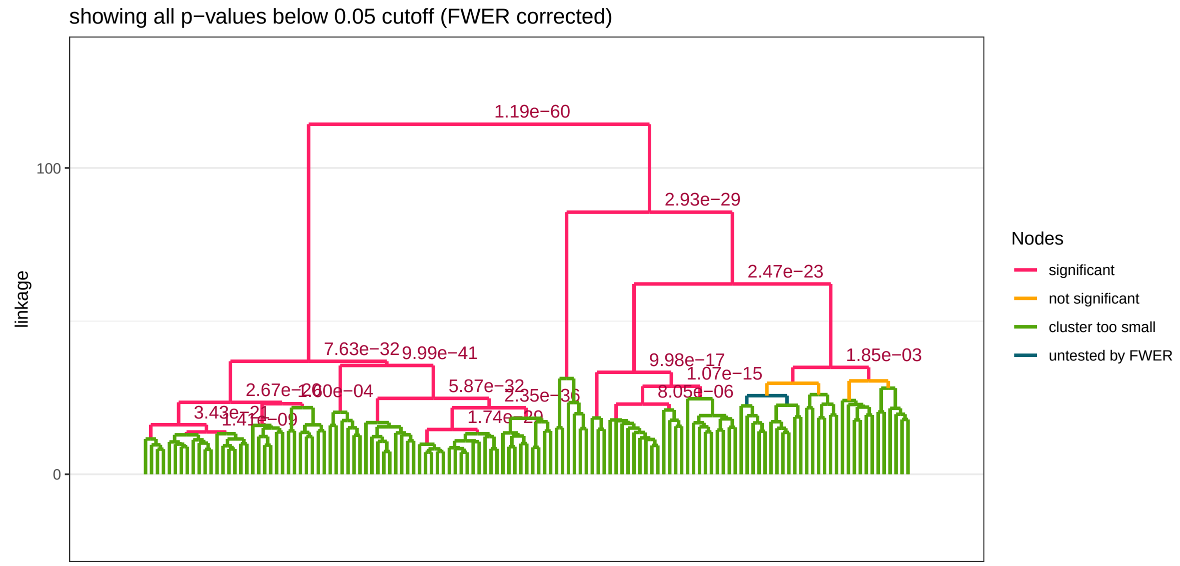

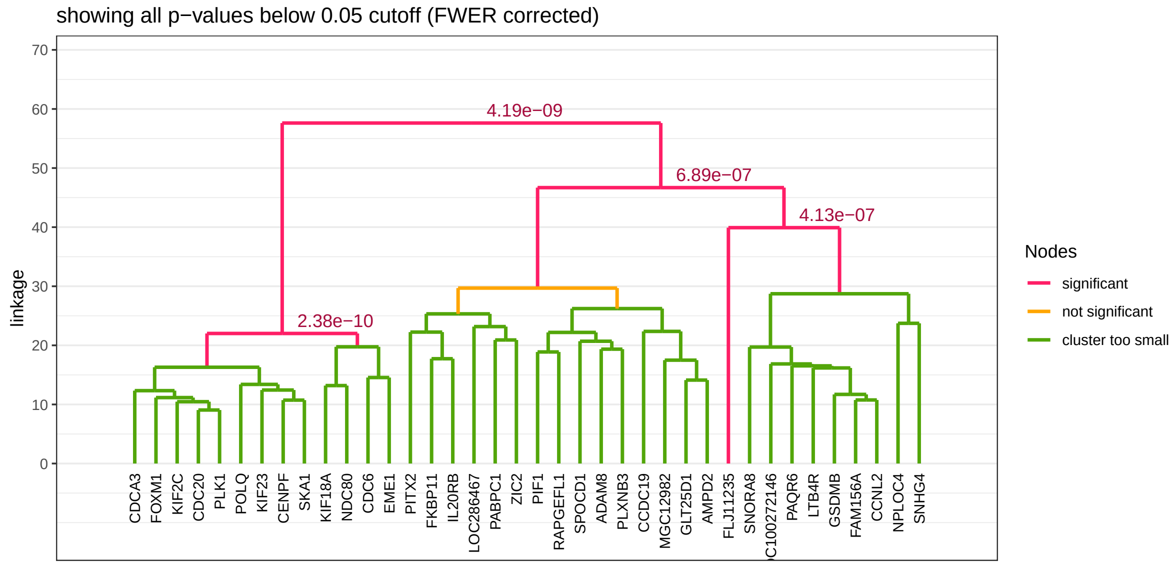

Supplementary Figure 23: **Significance Analysis of Hierarchical Clustering for HFS Genes (upper) and HiFIT Genes (lower) in the TCGA Cohort.** Significant nodes indicate that the genes grouped under these nodes exhibit differential expression, whereas non-significant nodes suggest similar expression patterns of genes across patients.

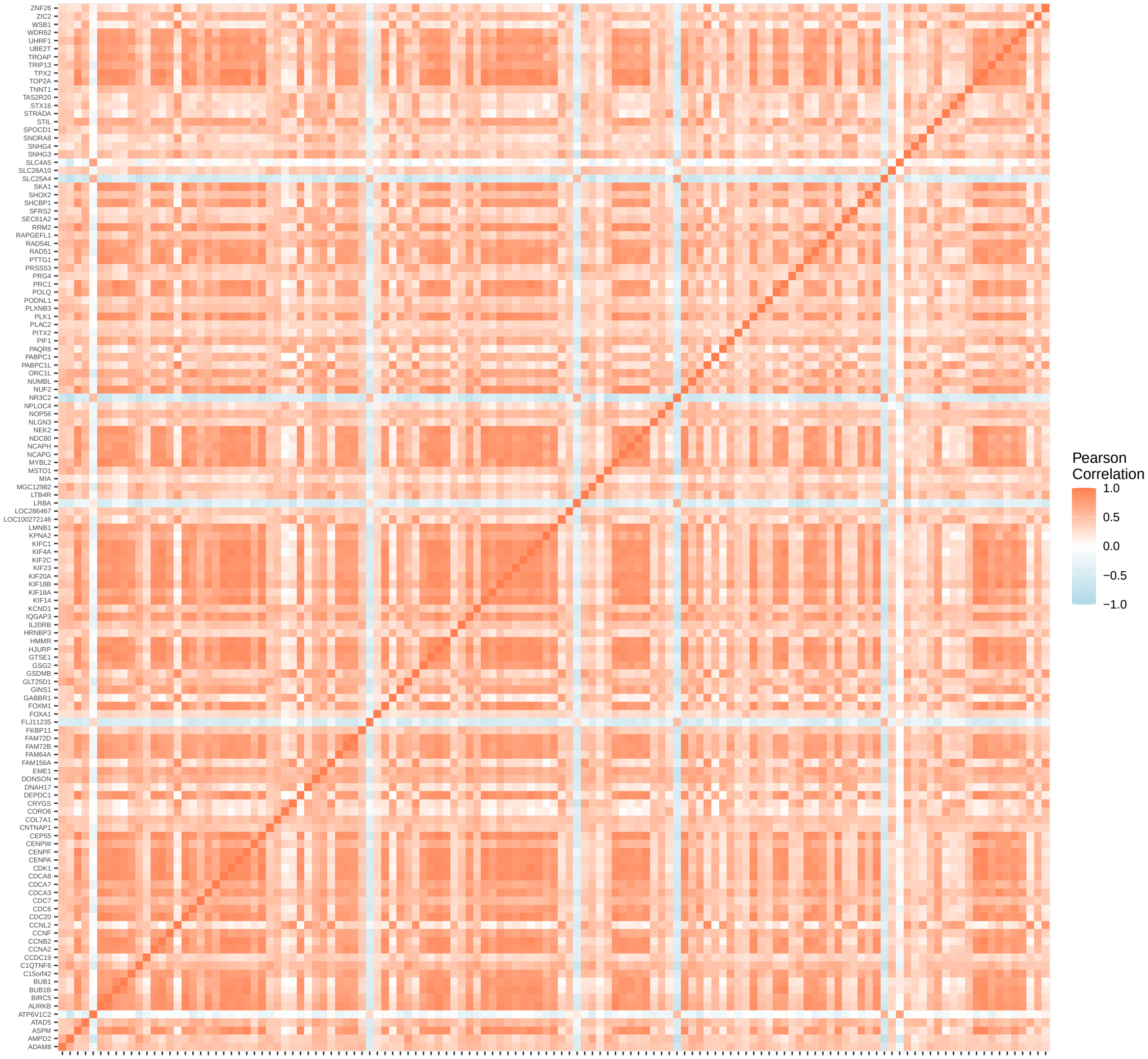

Supplementary Figure 24: **The Correlation Matrix of HFS Genes in the TCGA Cohort.**

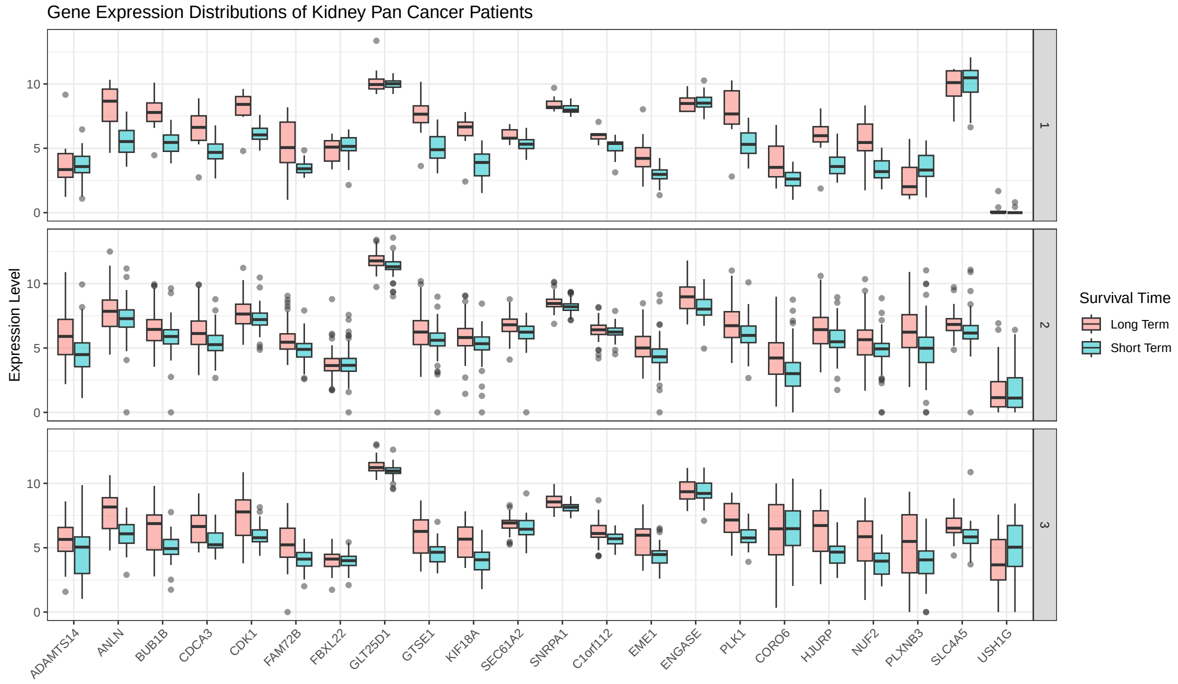

Supplementary Figure 25: **Association across Kidney Pan Cancer Survival, Gene Expression, and Histological Type in the TCGA cohort.** Each row represents a histological type.
